# Nonmalignant AR-positive prostate epithelial cells and cancer cells respond differently to androgen

**DOI:** 10.1530/ERC-22-0108

**Published:** 2022-10-10

**Authors:** Konsta Kukkonen, Bryn Autio-Kimura, Hanna Rauhala, Juha Kesseli, Matti Nykter, Leena Latonen, Tapio Visakorpi

**Affiliations:** 1 Faculty of Medicine and Health Technology, Tampere University and Tays Cancer Centre, Tampere University Hospital, Tampere, Finland; 2 Foundation for the Finnish Cancer Institute, Helsinki, Finland; 3 Institute of Biomedicine, University of Eastern Finland, Kuopio, Finland; 4 Fimlab Laboratories Ltd, Tampere, Finland

**Keywords:** prostate epithelium, prostate cancer, androgen receptor, androgen response, cell models

## Abstract

Prostate cancer research suffers from the lack of suitable models to study the role of normal cells in prostate carcinogenesis. To address this challenge, we developed a cell line model mimicking luminal prostate epithelial cells by modifying the immortalized prostate epithelial cell line RWPE-1 to constitutively express the androgen receptor (AR). RWPE-1-AR cells express known AR target genes, and exhibit coexpression of luminal and basal markers characteristic of transient amplifying cells, and an RNA signature resembling prostate luminal progenitor cells. Under unstimulated conditions, constitutive AR expression does not have a biologically significant effect on the proliferation of RWPE-1 cells, but when stimulated by androgens, growth is retarded. The transcriptional response of RWPE-1-AR cells to androgen stimulation involves suppression of the growth-related KRAS pathway and is thus markedly different from that of the prostate cancer cell line LNCaP and its derivative AR-overexpressing LNCaP-ARhi cells, in which growth- and cancer-related pathways are upregulated. Hence, the nonmalignant AR-positive RWPE-1-AR cell line model could be used to study the transformation of the prostate epithelium.

## Introduction

Prostate cancer (PCa) is a major cause of morbidity and mortality in the Western world (Ferlay *et al.* 2019). With the aging of the population, the incidence of PCa is on the rise, increasing the burden on the health care system. Large-scale sequencing studies have provided valuable information regarding the heterogeneity of the disease and have identified many potential targets for personalized medicine (Taylor *et al.* 2010, Barbieri *et al.* 2012, Abeshouse *et al.* 2015, Gundem *et al.* 2015, Fraser *et al.* 2017, Robinson *et al.* 2017, Armenia *et al.* 2018, Wedge *et al.* 2018). However, androgen receptor (AR) targeting remains the most effective therapeutic option for most patients with metastatic disease.

A major barrier in understanding prostate tumorigenesis is the lack of suitable models to study PCa driver alterations in a systematic manner. This stems from difficulties growing primary prostate epithelial cells in both standard cell culture conditions and in more specialized organoid culture systems in the laboratory (van Bokhoven *et al.* 2003, Sampson *et al.* 2013, Drost *et al.* 2016, Saeed *et al.* 2017). Thus, most studies on normal prostate epithelial cells and early tumorigenesis have been performed with mouse prostates. However, the normal mouse prostate gland differs clearly from the normal human prostate, and the mouse prostate does not spontaneously give rise to PCa (Ittmann 2018). On the other hand, human PCa cell lines are derived from treated metastatic PCa and thus have a high mutational burden. Consequently, the current models are suboptimal for the study of early prostate tumorigenesis. Prostate epithelial cells with luminal features have been particularly difficult to maintain for long periods in cell culture. Most nonmalignant prostate epithelial cell line models, such as EP156T and HPr-1 (Choo *et al.* 1999, Kogan *et al.* 2006, Olsen *et al.* 2016), resemble basal-type epithelial cells, which are AR negative. However, primary PCa cells are almost always AR positive, and they express AR target genes and luminal markers. Although which type of prostate epithelial cells (basal or luminal) give rise to PCa remains debatable, most recent studies suggest that PCa stem cells are AR positive (Wang *et al.* 2009, 2014, Germann *et al.* 2012, Guo *et al.* 2020).

Here, we used the premalignant and immortalized prostate cell line RWPE-1 (Bello *et al.* 1997) to establish a cell model representing luminal cells expressing AR, namely RWPE-1-AR. We characterized the growth properties of these cells and showed that they respond to androgen stimulus. By transcriptional characterization and comparison to the PCa cell lines LNCaP and LNCaP-ARhi, which mimic late-stage castration-resistant PCa, we show that the RWPE-1-AR cell line is useful as a nonmalignant, AR-positive prostate epithelial cell model for studying the transformation of the prostate epithelium.

## Materials and methods

### Cell lines

The RWPE-1 and LNCaP cell lines originated from the American Type Cell Culture Collection and were tested for authenticity using custom SNP panels (8 unique SNPs/cell line). EP156T cells were gift from Warda Rotter lab. RWPE-1 cells were cultured in K-SFM (Gibco/Thermo Fisher Scientific), except during the DHT stimulation experiments, for which they were cultured in phenol-red free RPMI-1640 (Lonza, Basel, Switzerland) supplemented with 5% V/V charcoal filtered FBS and 2 mM l-glut. EP156T cells were maintained in complete K-SFM supplemented with 5 ng/mL Na_2_SeO_4_ and 10 nM DHT, except after transduction, after which the DHT supplement was drawn. LNCaP cells and their derivatives LNCaP-pcDNA3.1 and -ARhi, established by us (Waltering *et al.* 2009), were maintained in RPMI-1640 with 10% V/V FBS and 2 mM l-glut, and for the DHT stimulation experiments in the same medium as the RWPE-1 cells except with 10% charcoal filtered FBS. Both cell lines were subcultured twice a week. The 293T cells used for virus packaging were cultured in D-MEM (high glucose) supplemented with 10% V/V FBS and 2 mM l-glut and subcultured 3 times a week. PC-3 cells used as positive control for soft agar assay were cultured in Ham’s F-12 supplemented with 10% V/V FBS and 2 mM l-glut and subcultured twice a week.

### AR transduction

The AR-containing vector pWPI-MCS-AR was cloned in-house and packaged with Delta9.81 and VSVg vectors in 293T cells. Virus-containing supernatant was concentrated using an ultracentrifuge at 80,000 *g* for 90 min. Viral titers were tested by Accuri C6 flow cytometry (BD, Franklin Lakes, NJ, USA), and multiplicity of infection = 2 was used to transduce RWPE-1 cells. The cells were enriched for green fluorescent protein (GFP) expression using FACSAria Fusion (BD) to facilitate clone isolation. Single cells were picked either using cloning rings or based on GFP under an epifluorescence microscope.

### Growth curves

For the DHT stimulation experiment, 1 × 10e4 cells, and for the non-DHT experiment, 2 × 10e3 cells were seeded into 48-well plates in K-SFM. The next day, alamarBlue or prestoBlue (Invitrogen/Thermo Fisher Scientific) premixed with hormone-stripped medium supplemented with 0, 1, 10, or 100 nM DHT or with K-SFM was changed to the cells for 2 h (alamarBlue used for DHT stimulations) or 30 min (prestoBlue used for K-SFM) on each day of measurement (different wells each day) for 7 days. Cell growth was measured in six (K-SFM growth curves) or eight (DHT-stimulations) replicates. Fluorescence was measured with an EnVision 2104 multilabel reader (PerkinElmer) with excitation 570 nm and emission 585 nm. Growth curve data were normalized to day 1 results, and differences in growth between conditions were assessed with one-way ANOVA and Tukey’s* post hoc* test with GraphPad Prism v5.02.

### Cell cycle analysis

Two experiments were performed. Cells were seeded into six-well plates in duplicate at the same density as in the DHT-stimulated growth assay. On the next day, hormone-stripped medium supplemented with 0, 1, 10, or 100 nM DHT or fresh K-SFM was changed to the cells. Seventy-two hours after the medium was changed, the cells were trypsinized, fixed, and stained according to the protocol provided by the manufacturer for the propidium iodide staining solution (Invitrogen/Thermo Fisher Scientific). Cells were run with a CytoFlex S flow cytometer (Beckman Coulter, Brea, CA, USA). Unstained samples from the first experiment were used to verify that GFP expressed from the AR vector did not bleed to the PI channel. The cell cycle data were analyzed with ModFit LT v5.0 (Verity Software House) using gating with PI-A/PI-H, followed by manual analysis with ploidy set to diploid and manually defined G1 and G2 peaks. Aggregates and debris were also modeled. Statistical differences in the cell cycle states between conditions were tested with GraphPad Prism v5.02 using one-way ANOVA followed by Tukey’s* post hoc* test.

### Analysis of apoptosis

Cells were grown as for the DHT-stimulated growth analysis, collected after 72 h, and stained for Annexin V using the eBioscience Annexin V Apoptosis Detection Kit APC (Invitrogen/Thermo Fisher Scientific) according to the manufacturer’s instructions. DAPI was used as the cell viability dye in the experiment. For the analysis, 15,000 events were recorded by the CytoFlex S flow cytometer. Compensations were recorded with unstained cells (GFP) and heat-killed RWPE-1 parental cells stained with Annexin V antibody or with DAPI, for the respective fluorophores, and applied before the analysis. Analysis was performed with CytExpert v2.4.

### Wound healing assay

Cells were seeded into 24-well plates in replicates from 2 to 8 wells/cell line in K-SFM. Three days after seeding, scratches were made on confluent cell layers using a p200 pipette tip. Cells were imaged every 3 h for 48 h Using an EVOS FL auto (Life Technologies/Thermo Fisher Scientific) imaging system with 4× magnification and phase contrast imaging. Image analysis was performed with Fiji/ImageJ v2.1.0/1.53c (Schindelin *et al.* 2015, Rueden *et al.* 2017) using the Wound_healing_size_tool custom plugin (Suarez-Arnedo *et al.* 2020) with standard parameters except for the variance window radius, which was 10 pixels. The scratch width in pixels was converted to micrometers based on the size of the scale bar in the image (1 pixel = 2222 µm). The results were averaged for each well before statistical analyses. Statistical testing was performed with two-way ANOVA followed by Bonferoni correction in GraphPad prism v5.02.

### Colony formation assay

Lower agar was prepared by casting 0.5% W/V Noble agar (Merck Millipore) diluted into normal growth medium of the respective cell line into 6-well plates. After 30 min incubation at room temperature (RT) cells of each cell line were seeded to the wells in 0.35% W/V Noble agar in 5000 cells/well in 3 replicates for each experiment. Cells were then left to grow for 3 weeks during which fresh medium was added to the wells twice a week. At the endpoint, excess medium was removed, and the cells were stained with 0.01% crystal violet and imaged with 4× magnification using EVOS FL auto at 3 randomly selected focus points from each well. Colonies in each focus point were then manually counted.

### Western blot

Cells were scraped into PBS and spun down at 500 ***g***. A previously published protocol (Abmayr *et al.* 2006) was used for nuclear protein extraction. The protein concentrations of the nuclear extracts were measured with a DC protein assay (BioRad). Samples were diluted with 3× Blue Protein Loading Dye (New England Biolabs, Ipswich, MA, USA) and boiled at 95°C for 3 min. The samples (15 µg) were electrophoresed on a 12.5% V/V SDS–PAGE gel and then transferred onto an Immobilon-P PVDF membrane (Merck Millipore) at 50 V for 14 h at +4°C. Membranes were blocked with 3% W/V BSA in PBS for 1 h at RT and probed with primary antibody against AR, ERK1/2, pERK1/2, CDK4, lamin B, or β-tubulin (Supplementary Table 15) 1 h at RT. The membranes were washed with TBST (20 mM Tris, 150 mM NaCl and 0.01% V/V Tween-20 at pH 7.6), incubated with secondary antibodies (1:5000 rabbit α-mouse-horse radish peroxidase (HRP) or 1:5000 swine α-rabbit-HRP) for 1 h at RT and washed before being detected with Clarity Western ECL reagent (BioRad). Quantitation of the AR and lamin-B protein bands from scanned films was performed with ImageJ v1.53c.

### Immunofluorescence staining and epifluorescence microscopy

For AR staining, the cells were seeded on coverslips in K-SFM, and the following day, the medium was replaced with hormone-stripped medium for 72 h. Then, 0, 1, or 100 nM DHT was added to the cells for 2 h. For Ki-67 staining, cells were seeded on coverslips in K-SFM and cultured as described for the DHT-stimulated growth curves for 72 h. Cells were fixed with 4% V/V formaldehyde for 30 min at RT (16% stock from Pierce/Thermo Fisher Scientific), permeabilized with 0.5% V/V NP-40 substitute for 5 min at RT, and blocked with PBS containing 3% W/V BSA for 10 min at RT. The coverslips were then incubated with primary antibody for AR or Ki-67 (Supplementary Table 15) for 1 h at 37°C, washed and incubated with a goat α-mouse AlexaFluor 568 secondary antibody (Invitrogen/Thermo Fisher Scientific) diluted 1:200. After washing, the coverslips were stained with DAPI and washed again. The coverslips were then embedded onto objective glasses with Vectashield (Vectorlabs, Burlingame, CA, USA) or Fluorescence mounting medium (Dako/Agilent Technologies).

Imaging was performed using an Olympus IX51 with a 20× magnification and constant exposure time for all samples. The images were analyzed using ImageJ v1.53c. For image processing, contrasts were adjusted to improve feature visibility.

### RNA sample preparation

Cells were hormone-stripped for 72 h, followed by 24 h (for RT-qPCR) or 4 h (for RNA-seq) of stimulation with 0, 1, or 100 nM DHT. Samples were collected in TRIzol reagent (Thermo Fisher Scientific). For RNA-seq, samples were additionally DNAse-treated on columns with DNAse I (Qiagen) during RNEasy (Qiagen) column purification. Sample quality was assessed using a Fragment Analyzer with Standard Sensitivity RNA Analysis kit (AATI/Agilent). Library preparation was performed using standard polyA enrichment and sequenced with a Novaseq6000 (Illumina, San Diego, CA, USA; at Novogene facility in Hong Kong).

### Reverse-transcription quantitative polymerase chain reaction

The RNAs (1000 ng/sample) were reverse transcribed with Maxima RT (Invitrogen/Thermo Fisher Scientific) according to the manufacturer’s protocol using random hexamer primers. The expression of AR target genes was then quantified with gene-specific primers (Supplementary Table 16) using 2× SYBR green master mix (Thermo Fisher Scientific) and CFX384 Real-Time PCR Detection System (BioRad) or with CFX opus 96 (BioRad) and normalized to the housekeeping gene (TBP) using the 2^−ΔΔCt^ method (Livak & Schmittgen 2001).

### Sequencing data analysis

The quality of raw RNA-seq reads was evaluated using FastQC v0.11.8 (https://www.bioinformatics.babraham.ac.uk/projects/fastqc/), and the reads were trimmed with Trim Galore! v.0.6.5 (https://github.com/FelixKrueger/TrimGalore) using the parameters --phred33 --stringency 5 --paired. Reads were aligned to GRCh38 using STAR v2.71a (Dobin *et al.* 2013) and indexed with Samtools v1.8 (Li *et al.* 2009). Reads were also pseudoaligned to Gencode comprehensive gene annotation on the primary assembly v30 (Frankish *et al.* 2019) with Kallisto v0.45.0 (Bray *et al.* 2016). The transcript counts were analyzed for DE using the DESeq2 R package (Love *et al.* 2014) and shrunk to reduce noise with apeglm (Zhu *et al.* 2019) R package. GSEA with hallmark gene sets (Liberzon *et al.* 2015) and KEGG pathway (Kanehisa *et al.* 2017) enrichment analyses were performed with the R packages fgsea (Korotkevich *et al.* 2021) and GAGE (Luo *et al.* 2009), respectively. Databases were accessed using the biomaRt R package (Durinck *et al.* 2009).

### Results

#### Establishment of AR-expressing RWPE-1 cells

**Figure 1:**
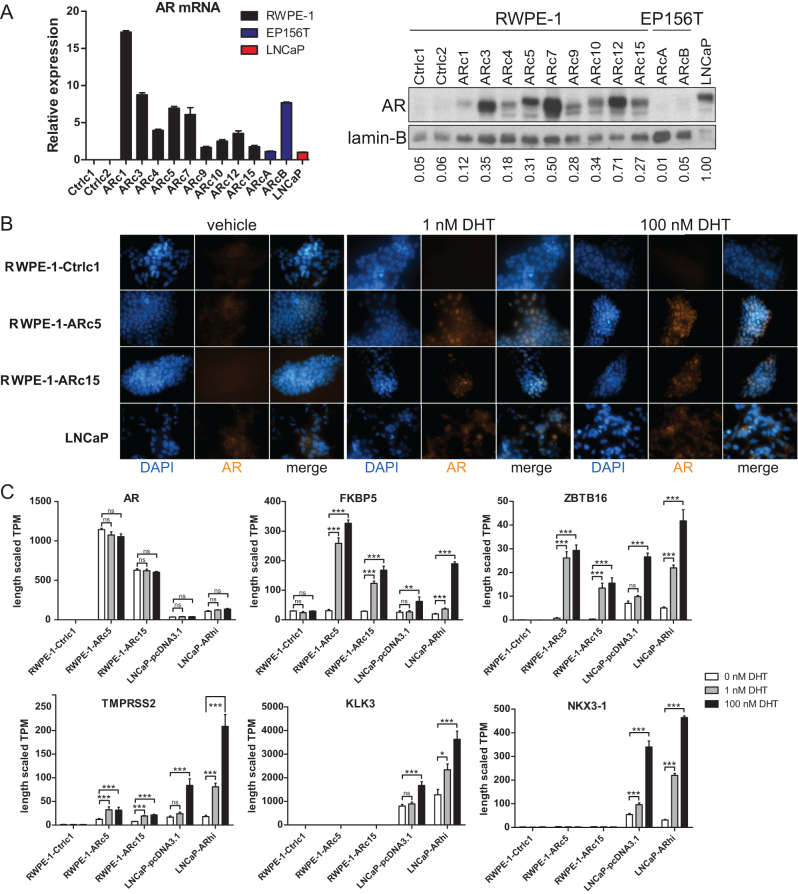
Characterization of RWPE-1-AR cells. (A) Left: AR mRNA level expression in the candidate AR clones as measured by RT-qPCR, mean, and s.e.m. of three technical replicates are shown. Right: AR protein level expression in candidate AR clones. Lamin B was used as a loading control. LNCaP is included as a positive control. Quantitation result relative to LNCaP is shown below the loading control. (B) The nuclear localization of AR after 2 h DHT stimulation with indicated DHT concentrations. (C) The expression of AR and its target genes FKBP5, ZBTB16, TMPRSS2, KLK3, and NKX3-1 after 4 h stimulation with DHT (0, 1, or 100 nM) as quantified by mRNA-sequencing. mRNA-sequencing data represent mean and s.e.m. of three technical replicates. Benjamini–Hochberg (B–H) adjusted *P*-values from Wald test for gene expression between vehicle and DHT-treated cells are indicated for all cell lines with *<0.05, **<0.01, and ***<0.001, ns, not significant.

To establish stable AR expression in nonmalignant prostate epithelial cells, we transduced RWPE-1 and EP156T (Kogan *et al.* 2006) cells with an *AR*-carrying lentiviral vector. Several AR-expressing and control clones were isolated, and the AR levels were measured by RT-qPCR and Western blotting (Fig. 1A). All studied AR-transduced RWPE-1 clones presented robust AR expression comparable to that in the LNCaP cell line, but no detectable AR protein was present in the EP156T clones despite robust expression at the mRNA level. Based on the favorable responses to dihydrotestosterone (DHT) in the initial screening using RT-qPCR (Supplementary Figure 1, see section on supplementary materials given at the end of this article), we selected two clones (RWPE-1-ARc5 and RWPE-1-ARc15) for further characterization.

Next, we analyzed the functionality of the constitutively expressed AR in the selected RWPE-1-AR and control clones. The keratinocyte serum-free medium (K-SFM) that is the standard medium for culturing RWPE-1 cells contains phenol red, which can act as a weak steroid hormone and interfere with the quantitation of androgen-regulated gene expression (Berthois *et al.* 1986). In addition, the bovine pituitary extract used as a supplement in the medium may contain residual androgens. Thus, we used phenol red-free RPMI-1640 supplemented with 5% charcoal-stripped FBS and 2 mM l-glutamine as the hormone starvation medium for the RWPE-1 cells throughout the study. We first confirmed the stimulation-dependent translocation of AR from the cytoplasm to the nucleus using immunofluorescence staining of hormone-deprived and hormone-stimulated cells. In RWPE-1-AR cells, the nuclear translocation of AR was obvious after 2 h even at low levels (1 nM) of DHT but was not observed in its absence (Fig. 1B). The AR-negative control cells showed no AR, and in the LNCaP cells, DHT augmented the nuclear accumulation of AR, as expected.

To study the effect of DHT stimulation on the expression of AR target genes, we performed mRNA-sequencing (RNA-seq) of AR-expressing RWPE-1 clones stimulated with 0, 1, or 100 nM DHT for 4 h. In addition, we RNA-sequenced DHT-stimulated LNCaP-pcDNA3.1 control cells and AR-overexpressing LNCaP-ARhi cells, which we previously established (Waltering *et al.* 2009). Known AR target genes, such as *FKBP5* encoding prolyl isomerase 5, *ZBTB16* encoding zinc finger and BTB domain-containing protein 16, and *TMPRSS2* encoding transmembrane serine protease 2, were significantly induced in RWPE-1-AR, whereas others, such as *KLK3* encoding PSA and *NKX3-1* encoding NK3 homeobox 1, were not (Fig. 1C). These effects were observed at physiologically low levels of androgen (1 nM DHT) and retained in superphysiological (100 nM DHT) concentrations. The results indicate that while AR is transcriptionally active upon androgen stimulation in RWPE-1-AR cells, other factors in addition to AR are likely required to induce the expression of certain AR target genes.

#### RWPE-1-AR cells have a luminal progenitor-like transcription pattern

**Figure 2:**
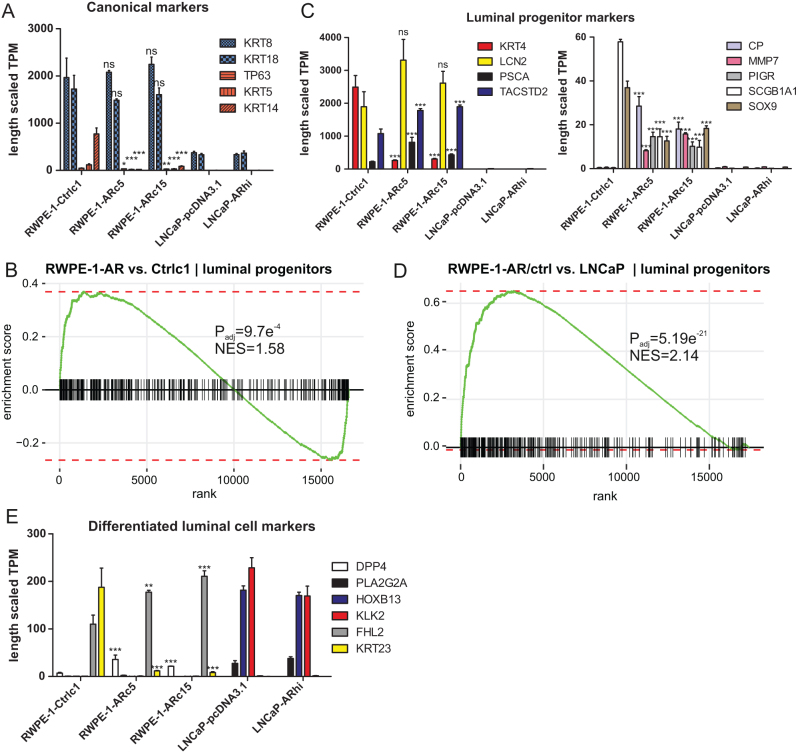
The expression of lineage-specific markers in RWPE-1 and LNCaP cells. (A) The expression of canonical luminal (KRT8, KRT18) and basal (TP63, KRT5, KRT14) marker genes in 0 nM DHT. Mean of three replicates ± s.e.m. is shown. B–H adjusted *P*-values from Wald test for difference in gene expression in comparison to Ctrlc1 are indicated for AR-expressing RWPE-1 clones with *P < 0.05, **P < 0.01, and ***P < 0.001, ns, not significant. (B) GSEA of luminal progenitor cell (luminal-C) signature between RWPE-1-AR and RWPE-1-Ctrlc1. Luminal progenitor cell signature from Guo *et al.* (2020). *P*_adj_ is B–H adjusted *P*-value from fgsea multilevel. NES, normalized enrichment score. (C) The expression of the highest-ranking luminal progenitor marker genes that are expressed in our dataset. Genes with high expression on the left and genes with lower expression on the right. Marker genes from the studies by Henry *et al.* (2018), Karthaus *et al.* (2020), and Guo *et al.* (2020). Statistical tests as in panel A. (D) GSEA of luminal progenitor signatures between RWPE-1-AR/ctrl (all samples) and LNCaP-pcDNA3.1/ARhi (all samples). (E) The expression of the highest-ranking differentiated luminal cell marker genes that are expressed in our dataset. Marker genes from the studies by Henry *et al.* (2018), Karthaus *et al.* (2020), and Guo *et al.* (2020). Statistical tests as in panel A.

Next, we wanted to study the effects of AR activation on the differentiation of RWPE-1 cells. RNA-seq showed that RWPE-1-AR and control cells expressed canonical lineage marker genes of both luminal (*KRT8*, *KRT18*) and basal (*TP63*, *KRT5* and *KRT14*) prostate epithelial cells (Fig. 2A) in the absence of androgens. The expression of both luminal and basal markers is characteristic of transit-amplifying cells. Interestingly, in RWPE-1-AR cells, the expression of luminal marker *KRT18* was slightly downregulated, and the expression of *TP63* and *KRT5* was upregulated following DHT stimulation, although the expression levels of the basal markers remained much lower than those of the luminal markers (Supplementary Fig. 2A). LNCaP cells lack basal cell markers, which is a general feature of PCa.

Recent single-cell analyses of human prostate have identified luminal progenitor cell populations resembling club and hillock cells of the lung epithelium (Henry *et al.* 2018). These cells survived castration and repopulated the prostate after the restoration of testosterone levels (Guo *et al.* 2020, Karthaus *et al.* 2020). Luminal progenitor cells were also found to be efficient targets for transformation in mice (Guo *et al.* 2020). As RWPE-1-AR cells are intended as a model for studying prostate transformation, we performed differential expression (DE) and gene set enrichment analysis (GSEA) between all RWPE-1-AR and control samples (all clones of the cell line in 0, 1, and 100 nM DHT) to determine the enrichment of the club and hillock cell signatures and the closely related luminal progenitor signature from the studies by Henry *et al.* (2018) and Guo *et al.* (2020). Although the RWPE-1-AR transcriptome did not have significant enrichment of club or hillock cell signatures, luminal progenitor signature was significantly enriched in comparison to control cell transcriptome (Fig. 2B). The expression of combined marker genes of club and luminal progenitor cells that were expressed in our dataset is shown for each cell line in Fig. 2C (0 nM DHT) and Supplementary Fig. 2B (all treatments). In particular, *PSCA* encoding prostate stem cell antigen and* TACSTD2* encoding tumor-associated calcium signal transducer 2 have increased expression in RWPE-1-AR cells in comparison to controls. Still, some luminal progenitor markers, such as *KRT4* encoding keratin type II cytoskeletal 4 and *SCGB1A1* encoding uteroglobin had higher expression in the controls than the RWPE-1-AR cells.

LNCaP cells, being true cancer cells, have very low expression of all the highest-ranking progenitor marker genes from the three studies mentioned earlier (Fig. 2C, Supplementary Fig. 2B), and consistent with this, the signatures of club, hillock, and luminal progenitor cells were significantly enriched to RWPE-1 transcriptome compared to LNCaP (Fig. 2D, Supplementary Fig. 2C-D).

Differentiated luminal cell signature defined by Henry *et al.* (2018) was not enriched between RWPE-1-AR and control cells consistent with the expression of basal markers in all clones or between all RWPE-1 and LNCaP samples consistent with the notion that cancer cells are undifferentiated by nature (Supplementary Fig. 2E-F). Indeed, the expression of the highest-ranking markers of the differentiated luminal cells was widely variable between the cell lines. For instance, the expression of *KRT23* encoding keratin type I cytoskeletal 23 was highest in RWPE-1-Ctrlc1, *DPP4* encoding dipeptidyl peptidase 4 in the RWPE-1-AR cells, and *HOXB13* encoding homeobox protein Hox-B13 and *KLK2* encoding kallikrein-2 in the two LNCaP cell lines (Fig. 2E, Supplementary Fig. 2G)

#### Stable expression of AR has little effect on RWPE-1 cell growth in standard conditions

**Figure 3:**
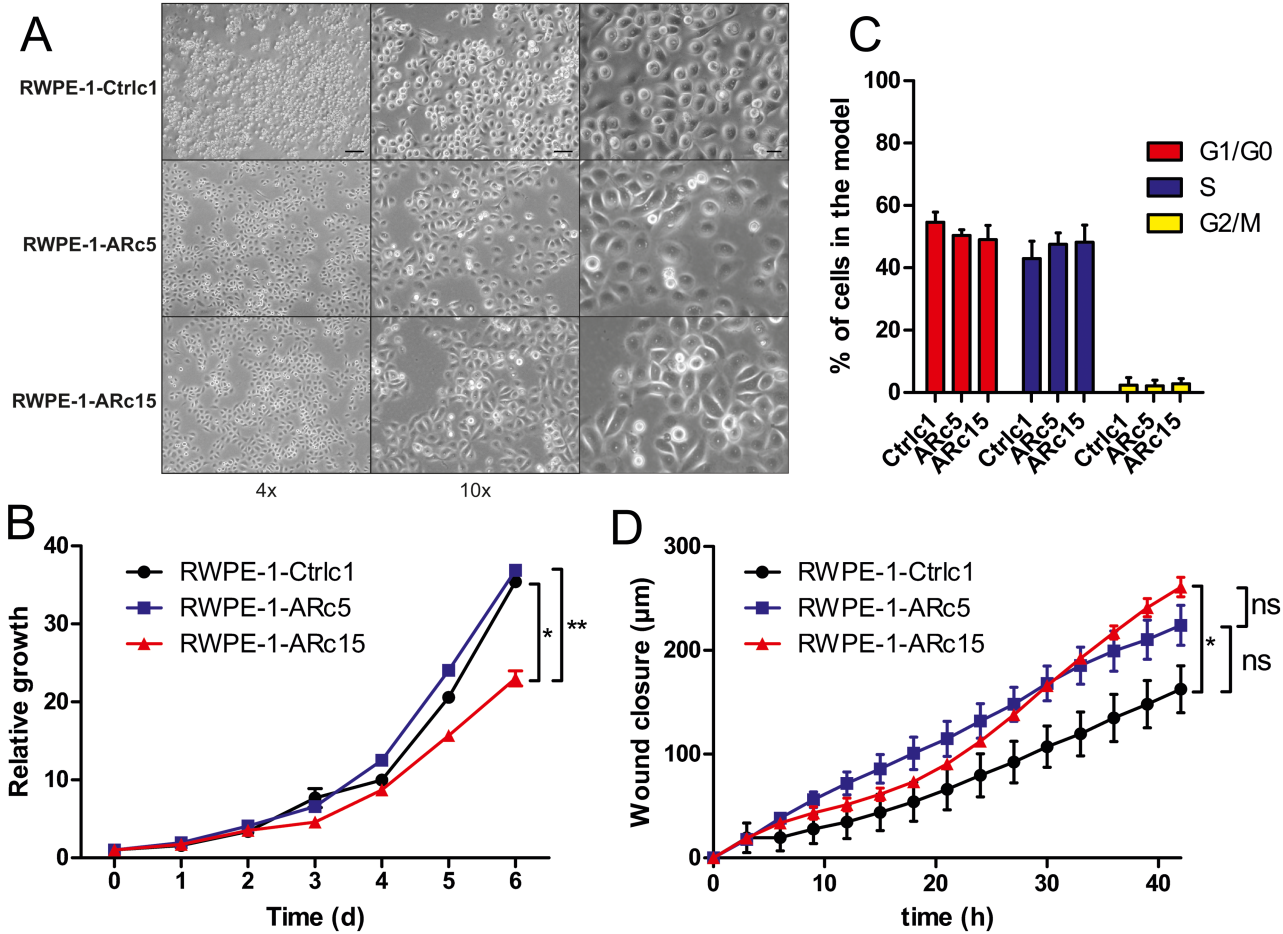
The growth and migratory characteristics of RWPE-1-AR and control cells in standard medium. (A) Images of cells grown in K-SFM for 48 h in 4×, 10×, and 20× magnifications. Scale bars 100, 50, and 20 µm, respectively. (B) The growth of RWPE-1-AR and control cells in K-SFM. The growth was measured daily up to 7 days using prestoBlue cell viability reagent. Datapoints show mean and s.e.m. for six replicates. C) The cell cycle analysis of RWPE-1-AR and control cells. Cells were grown for 72 h and stained with propidium iodide. Mean and s.e.m. of three replicates are shown. (D) Differences in the cell migration of RWPE-1-AR and control cells. Cells were cultured in K-SFM in six replicates for cell line. Statistical analyses were performed with Kruskal–Wallis test followed by Dunn’s multiple comparison test for the cell cycle stages, growth, and migration. *P < 0.05, **P < 0.01, ns, not significant. A full colour version of this figure is available at https://doi.org/10.1530/ERC-22-0108.

Next, we studied the effects of AR on the growth and other characteristics of RWPE-1 cells in their standard cell culture medium, K-SFM. The morphologies of the RWPE-1-AR and control clones in this medium differed slightly from each other. The AR-expressing clones RWPE-1-ARc5 and -ARc15 attached faster after passaging than the control clone, resulting in the controls having a more spherical shape 48 h after seeding (Fig. 3A). This difference, however, was reduced after 72 h (compare Fig. 3A to Supplementary Fig. 3A).

The growth assay showed similar growth rates for RWPE-1-ARc5 and the control clone, whereas RWPE-1-ARc15 grew slightly more slowly (Fig. 3B), indicating that the slower attachment of the control cells does not affect growth. When analyzing the cell cycle distribution using propidium iodide staining and flow cytometry, the RWPE-1 control cells had slightly more cells in G0/G1 phase than the RWPE-1-AR clones and conversely more cells were in S phase in the RWPE-1-AR clones (Fig. 3C, Supplementary Fig. 3B). However, the difference was not statistically significant.

Finally, we analyzed the motility and malignant properties of the RWPE-1-AR and control cells grown in K-SFM using a wound healing assay and colony formation assay. In the wound healing assay, AR-expressing RWPE-1 cells migrated more than the control cells although only the difference between ARc15 and the control clone reached statistical significance (Fig. 3D, Supplementary Fig. 3C). In the colony formation assay, none of the RWPE-1 cell lines formed any colonies, while PC-3 cells used as a positive control formed multiple colonies (Supplementary Fig. 3D), indicating that the RWPE-1 cells have kept their untransformed state after restoration of AR expression.

#### Effects of androgen on the growth of RWPE-1-AR cells

**Figure 4:**
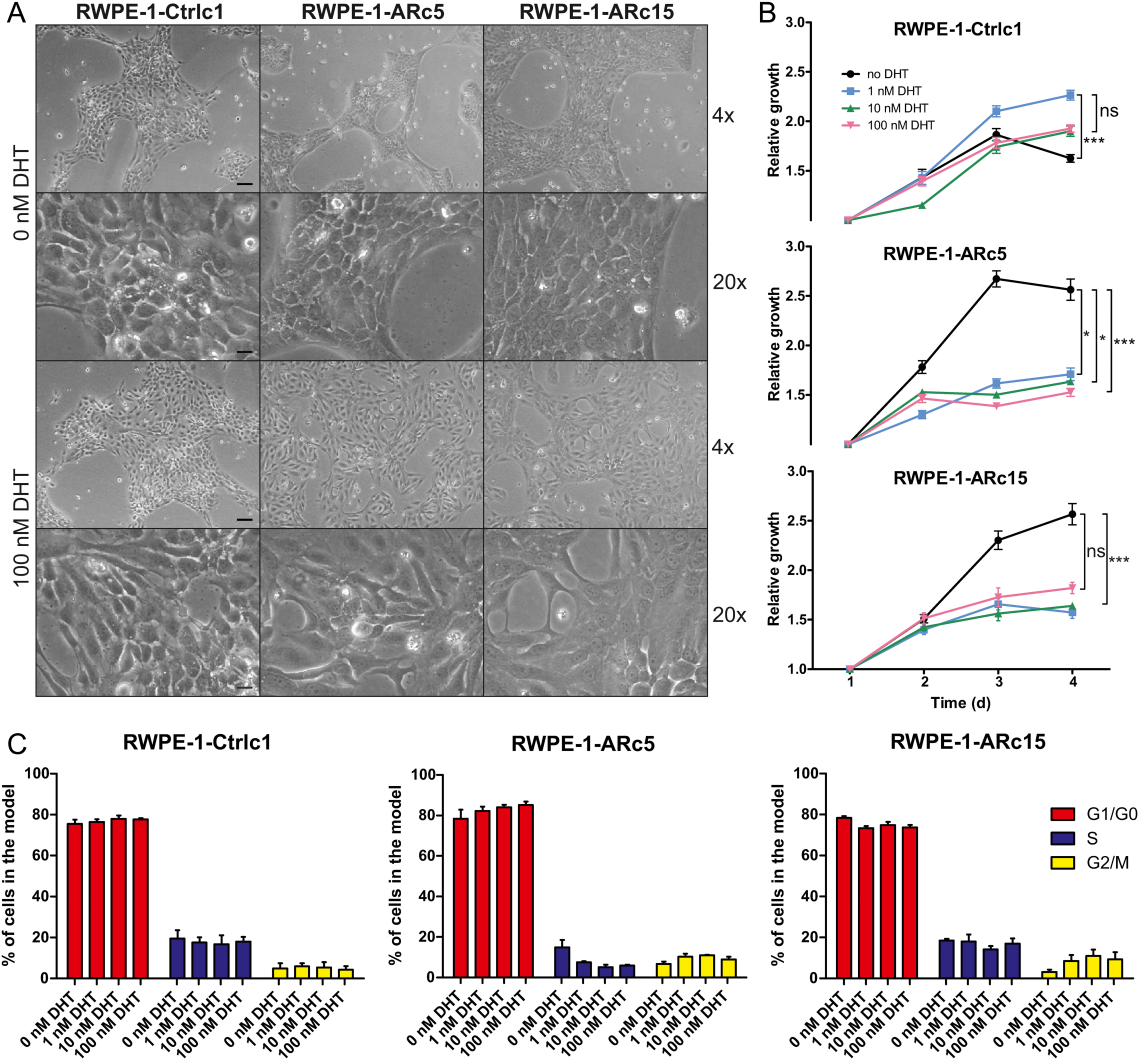
The effects of androgens on growth and cell cycle of RWPE-1-AR and control cells. (A) The morphology of RWPE-1-AR and control clones after 3 days exposure to 0 or 100 nM DHT. Scale bars 100 µm for 4× and 20 µm for 20× magnification. (B) The growth of RWPE-1-AR and control cells under stimulation with varying concentrations (0 nM, 1, 10, and 100 nM) of DHT. The growth was measured daily for 7 days using alamarBlue cell viability reagent. Results for four first days are shown, as the signal in all samples started to decrease after that. Datapoints show mean and s.e.m. for eight replicates. (C) The cell cycle analysis of RWPE-1-AR and control cells. The same protocol for culture was used for the growth curves (the same DHT concentrations, 72 h endpoint), after which cells were stained with propidium iodide. Mean and s.e.m. of three replicates are shown. Statistical analyses were performed for growth assay and cell cycles using Kruskal–Wallis test followed by Dunn’s test for multiple comparisons. *P*-values for growth assay are indicated for comparison between DHT-treated and vehicle treated cells. None of the comparisons for cell cycle analyses were significant. ***P < 0.001, *P < 0.05, ns, not significant.

To address the androgen-induced functions of AR in RWPE-1-AR cells, we next studied the growth characteristics of the clones upon stimulation with DHT. In the hormone-stripped medium, the RWPE-1 cells form tightly packed islands of cells (Fig. 4A) and grow more slowly than in their standard medium (compare Fig. 3B and Fig. 4B). These effects have been previously documented with PrEC and 957E/hTERT cells and were attributed to the high calcium content of the mediums such as RPMI-1640 used for culture of cancer cell lines (Dalrymple *et al.* 2005, Litvinov *et al.* 2006). Despite the effects caused by the RPMI medium, the growth of ARc5 and ARc15 was consistently reduced by DHT at all concentrations tested (1, 10, and 100 nM) (Fig. 4B). This is in line with previous findings in other nonmalignant prostate epithelial cells including PrEC-AR and HPr-1AR (Ling *et al.* 2001, Antony *et al.* 2014). The effect of DHT on growth rate was accompanied by marked changes in cell morphology and distribution pattern in the RWPE-1-AR cells, but not in the control cells. When stimulated with DHT, the RWPE-1-AR cells did not form as dense monolayers as they did under unstimulated conditions and had more protrusions such as filopodia (Fig. 4A).

To determine whether the growth retardation upon DHT treatment can be explained by changes in cell cycle distribution in the RWPE-1-AR clones, we performed cell cycle analysis (Fig. 4C, Supplementary Fig. 4A). The use of stripped medium increased the proportion of cells in G1/G0 in comparison to that of cells grown in K-SFM in all clones (from approximately 50–80%, compare Fig. 3C and 4C). However, we did not detect significant DHT-induced changes in the cell cycle distribution of these cells, nor were there systematic significant differences to the cell cycle distribution in comparison to the control cells. Cyclin-dependent kinase 4 (CDK4) expression is involved in G1/S transition. Expression of CDK4 was measured in cells treated with 0, 1, or 100 nM DHT for 72 h by Western blot (Supplementary Fig. 5A), but no systematic changes in DHT compared to the untreated cells were found. However, CDK4 expression was significantly higher in the cells cultured in K-SFM (Supplementary Fig. 5A) as expected based on the cell cycle analysis. To further study whether the proportion of actively replicating cells was altered by DHT, we performed immunostaining for the proliferation marker Ki-67 after stimulation with vehicle, 1 nM DHT, or 100 nM DHT (Supplementary Fig. 5B). While the ARc5 cells treated with 100 nM DHT were less positive for Ki-67 than the cells treated with no or 1 nM DHT, the DHT treatment had no apparent effect on Ki-67 positivity in Ctrlc1 or ARc15 (Supplementary Fig. 5B). In LNCaP cells, DHT slightly increased the Ki-67 staining intensity (Supplementary Fig. 5B). These results show that alterations in cell cycle distribution or fraction of actively proliferating cells cannot fully explain the growth changes observed in RWPE-1-AR cells upon DHT stimulation.

As the cells visibly suffered from the nonstandard medium used for the DHT stimulation experiments, we next investigated whether increased cell death could explain the observed growth retardation in RWPE-1-AR cells. Analysis of proportions of sub-G1 cells among the PI-stained cells from cell cycle analysis revealed a small trend of DHT-induced cell death in RWPE-1-AR cells, but the result was not statistically significant (Supplementary Fig. 5C). To specifically assess the extent of apoptosis under these conditions, we performed Annexin V staining. Overall, the proportion of early apoptotic cells (Annexin V^+^/DAPI^−^) was very low (<2% of total cells) in all samples, indicating that apoptosis does not account for the growth reduction in response to DHT in RWPE-1-AR cells (data not shown). However, by observing the proportion of nonpermeabilized DAPI stained cells, there was a clear DHT-dose-dependent increase in late apoptosis in ARc5 cells, while the trend was absent in ARc15 and Ctrlc1 cells (Supplementary Fig. 5D).

#### Androgens induce marked changes in the transcriptome of RWPE-1-AR cells

**Figure 5:**
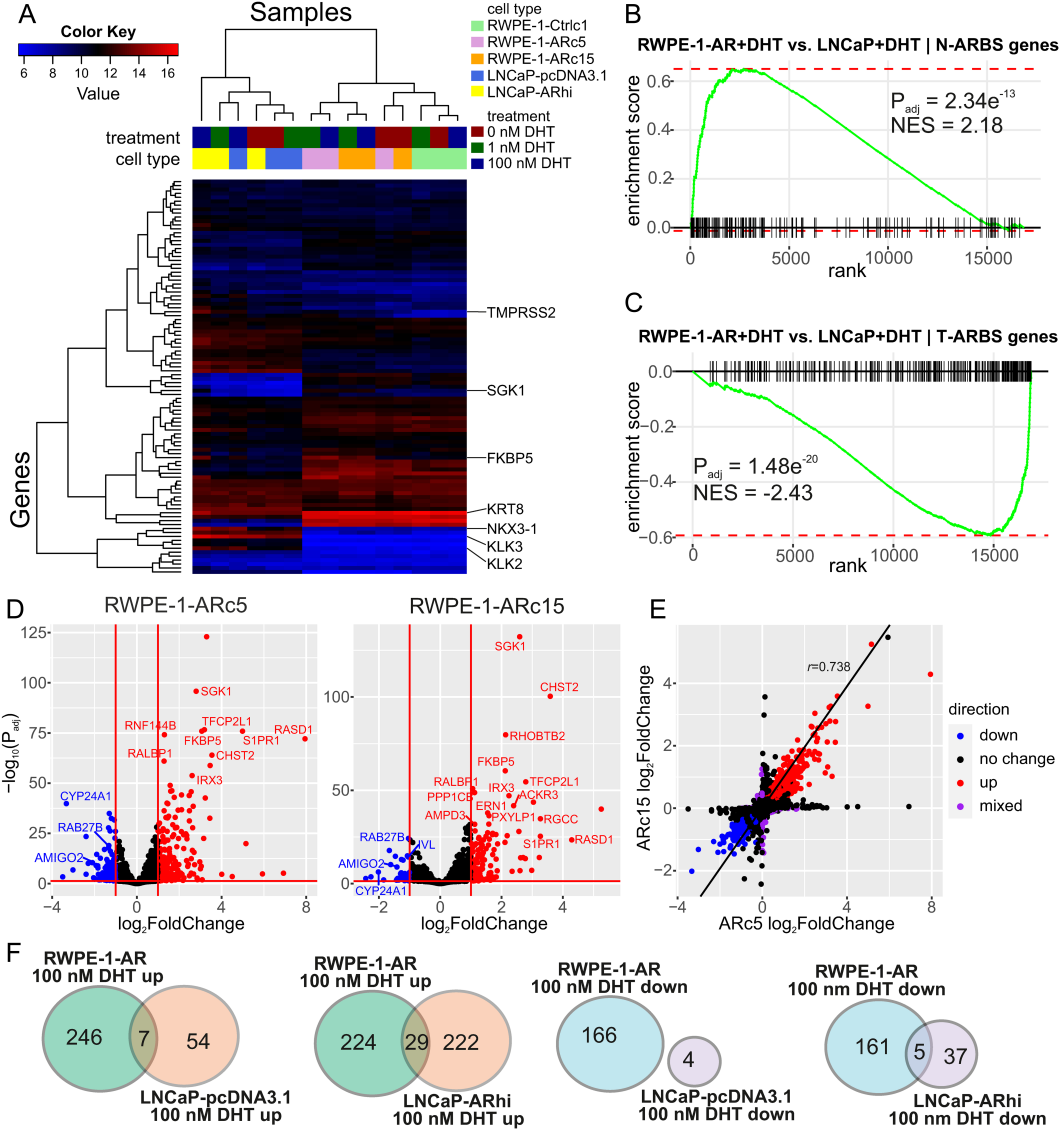
The cell type-specific transcriptomic responses to DHT stimulation. (A) A heatmap based on the HALLMARK ANDROGEN RESPONSE gene set. Classical AR target genes are marked on the right. Each sample shows the mean of three replicates. (B) GSEA of N-ARBS genes (genes downregulated in tumor vs normal tissue and within 50 kb of AR-binding site) between DHT-treated RWPE-1-AR cells and LNCaP cells. (C) GSEA of T-ARBS genes (genes upregulated in tumor vs normal and within 50 kb of AR-binding site) between DHT-treated RWPE-1-AR cells and LNCaP cells. Gene lists for N-ARBS and T-ARBS from study by Pomerantz *et al.* (2015). *P*-values in GSEA plots are multiple testing adjusted (B–H) values from fgsea multilevel. NES, normalized enrichment score. (D) Differential expression following DHT stimulation in RWPE-1-ARc5 and -ARc15 cells. Significantly upregulated genes are shown in red and downregulated in blue. The horizontal line indicates the threshold of statistical significance (B–H adjusted *P*-value from Wald test < 0.05) and the vertical lines log_2_fold change (log_2_fc) of −1 and 1. Some genes that are mutually regulated in both clones are labelled. (E) The correlation of the log_2_fcs of the two AR clones following DHT stimulation. The genes reaching statistically significant upregulation (red) and downregulation (blue) in both cell lines. Genes with mixed expression change (statistically significant up- or downregulation in one cell line and other direction of change in the other cell line) are shown in purple. (F) Comparison of mutually upregulated (*P*_adj_ < 0.05, log_2_fc > 1) and downregulated (*P*_adj_ < 0.05, log_2_fc < −1) genes in RWPE-1-AR and LNCaP-pcDNA3.1 and -ARhi cells at 100 vs 0 nM DHT.

Next, we focused on the gene expression changes related to the activation of AR using our RNA-seq dataset. First, we used unsupervised hierarchical clustering of the HALLMARK ANDROGEN RESPONSE gene set to cluster the cell lines based on their transcriptional response to DHT (Fig. 5A). The RWPE-1 and LNCaP cells formed their own separate clusters that highlight the differences in the androgen response between nonmalignant and cancer cells. Within the cell line subclusters, further differences in androgen responses are visible. In the RWPE-1-AR/control subcluster, Ctrlc1 cells form their own distinct cluster. The RWPE-1-AR cells treated with vehicle clustered near these controls, showing that without androgen stimulation AR target genes are expressed as in the controls. The DHT-treated ARc5 and ARc15 form their own clusters, but not in a dose-dependent manner, showing that AR activation does not change dramatically from 1 to 100 nM DHT in these cells. In the LNCaP subcluster, vehicle-treated cells clustered together. Interestingly, the 1 nM DHT-treated LNCaP-pcDNA3.1 cells also clustered with the vehicle-treated cells confirming our previous findings (Waltering *et al.* 2009) that these cells respond poorly to low levels of androgens. In contrast, LNCaP-ARhi cells treated with 1 nM DHT clustered with the 100 nM DHT-treated LNCaP-pcDNA3.1 and LNCaP-ARhi cells, consistent with their higher sensitivity to low DHT concentrations as we have previously shown (Waltering *et al.* 2009, Urbanucci *et al.* 2012).

Similar to the hierarchical clustering, principal component (PC) analysis separated RWPE-1-AR/control cells from the LNCaP cells by PC1 and further separated the AR-expressing RWPE-1 cells from the controls by PC2 (Supplementary Fig. 6A). When subjecting the top 100 genes having the most effect on PC2 to GO-enrichment analysis, many terms related to differentiation, development, peptidase activity, and immune responses were among the most significantly enriched terms (such as keratinocyte differentiation (GO:0030216; FDR 5.71e-06), negative regulation of peptidase activity (GO:0010466; FDR 5.03e-04), and antimicrobial humoral response (GO:0019730; FDR 5.26e-04)). To further study the difference between RWPE-1-AR and control cells, we performed differential gene expression (DE) analysis separately to AR clones and the control clone in all studied DHT concentrations. Of the 100 most significantly DE genes in each condition, between 50 and 61 were shared between both AR clones (ARc5 vs Ctrlc1 and ARc15 vs Ctrlc1) (Supplementary Tables 1–3). As expected, *AR* and its target gene *TMPRSS2* were on the top genes upregulated in the comparisons of DHT-treated samples, whereas none of the AR target genes come up in the comparison at 0 nM DHT.

As the cells originating from normal and cancerous prostates have vastly different expression patterns, even after identical treatments, we assessed the androgen regulation in normal vs cancer axis using data from a study by Pomerantz *et al.* (2015), who identified genes that were significantly downregulated or upregulated in cancer compared to normal tissue and resided within 50 kb of AR-binding sites (ARBS) (Pomerantz *et al.* 2015). We performed DE analysis of DHT-stimulated RWPE-1-AR (both clones, 1 and 100 nM DHT) cells and DHT-stimulated LNCaP cells (both -pcDNA3.1 and -ARhi, 1 and 100 nM DHT) to compare their responses with GSEA. The genes with ARBS in proximity and higher expression in normal tissue (N-ARBS genes) had significantly higher enrichment in RWPE-1-AR samples than LNCaPs (Fig. 5B). Conversely, LNCaP cells had significantly higher enrichment of genes bound by AR and upregulated in tumors (T-ARBS genes) (Fig. 5C). This verifies that despite of having high AR expression, the RWPE-1-AR cells retained a gene expression phenotype resembling that of normal prostate tissue.

Next, we studied androgen-induced gene expression in the RWPE-1-ARc5 and -ARc15 clones by DE analysis. As the clustering based on androgen-regulated genes showed no great difference in activation of AR signaling with either 1 or 100 nM DHT in the RWPE-1-AR cells, we compared samples treated with 1 or 100 nM DHT to those treated with vehicle only. A total of 1397 and 1203 genes were significantly differentially expressed when comparing DHT-treated and vehicle-treated RWPE-1-ARc5 and RWPE-1-ARc15 cells, respectively (B–H adjusted *P* < 0.05) (Fig. 5D). A total of 733 of these genes (or 39.3% of significantly DE genes in this comparison) were shared between the 2 clones (same direction of expression change) (Fig. 5D). Of these, 491 were upregulated and 242 were downregulated. On the transcriptome-wide scale, the DHT vs no DHT gene expression fold changes of the 2 cell lines (RWPE-1-ARc5 and -ARc15) had a Pearson correlation coefficient of 0.738, indicating a strong correlation (Fig. 5E). In RWPE-1-Ctrlc1 cells, DHT did not have a significant effect on gene expression. Taken together, these results show that overall, the two AR-expressing RWPE-1 cell lines have marked and similar responses to androgen stimulation.

In LNCaP-pcDNA3.1, there were only 2 significantly upregulated genes (*NKX3-1* and *SLC45A3*, both prostate-associated genes) and 1 downregulated gene (ENSG00000286022) when comparing 1 and 0 nM DHT stimulation, whereas in LNCaP-ARhi, there were 104 and 14 upregulated and downregulated genes, respectively. In 100 vs 0 nM DHT, there were 182 upregulated and 49 downregulated genes in LNCaP-pcDNA3.1 cells and 598 up- and 261 downregulated genes in LNCaP-ARhi cells (Supplementary Fig. 6B). Compared to the RWPE-1-AR clones, the correlation coefficient between the two LNCaP clones was lower (*r* = 0.579 in LNCaP compared to *r* = 0.738 in RWPE-1-AR, Supplementary Fig. 6C and Fig. 5E), in concordance with the known increased sensitivity of LNCaP-ARhi cells to lower levels of androgen.

To directly compare the DHT responses in RWPE-1-AR and LNCaP, we extracted lists of up- and downregulated genes (with 2-fold increase or 0.5-fold decrease in expression and adjusted *P* < 0.05) and analyzed the overlap between the cell lines (Fig. 5F). Only 7 genes were mutually upregulated by 100 nM DHT in LNCaP-pcDNA3.1 and both RWPE-1-AR clones (Supplementary Table 4), and no genes were mutually downregulated in RWPE-1-AR and LNCaP-pcDNA3.1 cells. In LNCaP-ARhi and RWPE-1-AR cells, 29 genes were mutually upregulated by 100 vs 0 nM DHT (Supplementary Table 5). Both lists of upregulated genes contained known AR target genes, such as *FKBP5*, *ZBTB16,* and *TMPRSS2*, as well as other PCa-related genes, such as *NDRG1*, which has been shown to have metastasis-suppressing properties (Supplementary Tables 4–5). Five genes were mutually downregulated in RWPE-1-AR and LNCaP-ARhi in 100 vs 0 nM DHT, including transmembrane receptors and signaling molecules, such as TGF-β signaling regulator (*BAMBI*) and TNF-ligand (*TNFSF15*) (Supplementary Table 6). These results illustrate the context dependency of androgen-regulated gene expression. Even though a few genes are mutually induced despite widely different genomic backgrounds, most genes seem to be regulated in only specific genomic settings.

#### Androgens suppress growth signaling in RWPE-1-AR cells

**Figure 6:**
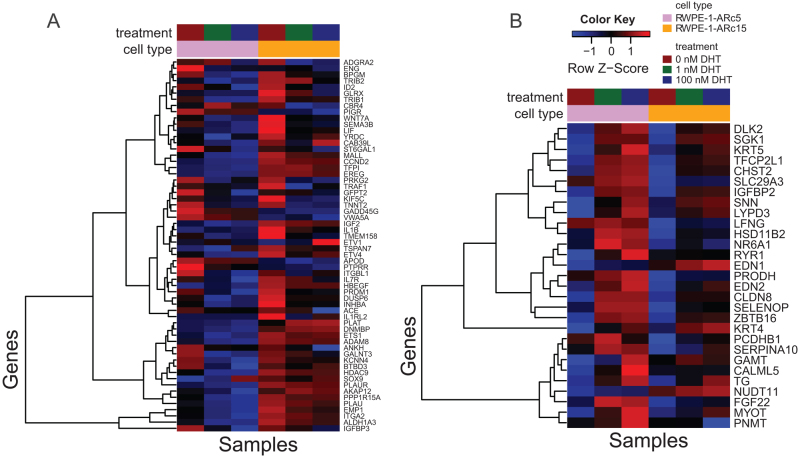
Suppression of KRAS signaling in RWPE-1-AR following DHT stimulation. (A) The leading-edge genes from the HALLMARK KRAS SIGNALING UP gene set. (B) The leading-edge genes from KRAS SIGNALING DN gene set.

To study the pathway level changes following androgen stimulation in RWPE-1-AR cells, we analyzed the DE data with GSEA for the combined DHT vs no DHT and found significant changes in 6 HALLMARK gene sets in ARc5 (Liberzon *et al.* 2015) and 8 HALLMARK gene sets in ARc15 (Table 1, separate tables for comparisons between 1 and 0 nM DHT and 100 and 0 nM DHT are shown in Supplementary Tables 7–10). Among the enriched gene sets in both AR-expressing clones were androgen response and, interestingly, hypoxia and interferon alfa and gamma responses as well as two KRAS signaling gene sets. KRAS signaling was reduced in DHT-treated cells (Fig. 6A– B), which may explain the growth reduction upon DHT stimulation (Fig. 4A). As KRAS signaling leads to activation of MAPK pathway, we quantified the amount of MAPK1 (ERK2) and MAPK3 (ERK1) using Western blot (Supplementary Fig. 7A). Amount of ERK1/2 was reduced in both RWPE-1-AR clones after 24 h DHT treatment and persisted at lower level in ARc15 after 48 h in a dose-dependent manner. We also assessed the expression of *EGFR*, one of the receptor tyrosine kinases activating the pathway leading to MAPK activation, from our gene expression data (Supplementary Fig. 7B). Interestingly in RWPE-1-ARc5, *EGFR* was significantly downregulated in 1 and 100 nM DHT, while the difference was not significant in ARc15 clone. Together these findings suggest that the growth of RWPE-1-AR cells is retarded in DHT-containing medium in dose-dependent manner due to decreased activity of KRAS–MAPK signaling pathway. No significantly enriched gene sets were found in RWPE-1-Ctrlc1 following DHT stimulation, further confirming the androgen insensitivity of these control cells.

**Table 1:** Effect of androgen stimulation on RWPE-1-AR. GSEA was run to DE analysis results of RWPE-1-ARc5 1 and 100 nM DHT samples vs RWPE-1-ARc5 0 nM DHT samples using fgsea simple with 1000 permutations. HALLMARK gene sets were used as the pool of gene sets. Results for the same comparison in RWPE-1-ARc15 cells are shown.

Pathway	*P*	*P*_adj_	ES	NES	nMoreExtreme	Size
**RWPE-1-ARc5 + DHT vs RWPE-1-ARc5 no DHT**						
HALLMARK_INTERFERON_ALPHA_RESPONSE	0.00298	0.0418	−0.588	−1.83	0	94
HALLMARK_KRAS_SIGNALING_DN	0.00148	0.0418	0.609	1.82	0	148
HALLMARK_INTERFERON_GAMMA_RESPONSE	0.00334	0.0418	−0.488	−1.66	0	182
HALLMARK_KRAS_SIGNALING_UP	0.00326	0.0418	−0.463	−1.57	0	161
HALLMARK_ANDROGEN_RESPONSE	0.00450	0.0450	0.577	1.64	2	94
HALLMARK_HYPOXIA	0.00575	0.0479	0.503	1.55	3	187
**RWPE-1-ARc15 + DHT vs RWPE-1-ARc15 no DHT**						
HALLMARK_KRAS_SIGNALING_DN	0.00141	0.0253	0.679	2.05	0	148
HALLMARK_ANDROGEN_RESPONSE	0.00152	0.0253	0.659	1.88	0	94
HALLMARK_INTERFERON_ALPHA_RESPONSE	0.00288	0.0253	−0.528	−1.,69	0	93
HALLMARK_ESTROGEN_RESPONSE_LATE	0.00279	0.0253	0.537	1.65	1	181
HALLMARK_HYPOXIA	0.00275	0.0253	0.521	1.61	1	187
HALLMARK_INTERFERON_GAMMA_RESPONSE	0.00355	0.0253	−0.451	−1.59	0	182
HALLMARK_KRAS_SIGNALING_UP	0.00347	0.0253	−0.448	−1.55	0	165
HALLMARK_TNFA_SIGNALING_VIA_NFKB	0.00714	0.0446	−0.417	−1.47	1	185

When comparing untreated RWPE-1-AR clones and the control clone, we identified upregulation of several immune response-related pathways (including interferon-gamma response) and hypoxia (Supplementary Table 11). Interestingly, the HALLMARK KRAS SIGNALING DN set had a negative enrichment score, indicating that in the setting of androgen starvation, the AR-expressing clones have a more active KRAS pathway than the control cells. The growth assay results are in concordance with this analysis, as the vehicle-treated AR cells grew faster than the controls (Fig. 4B).

In contrast to RWPE-1-AR, in LNCaP-pcDNA3.1 cells, only the androgen response pathway was borderline significant in GSEA (B–H adjusted *P* = 0.0614) when comparing 100 with 0 nM DHT (Supplementary Table 12). In LNCaP-ARhi, on the other hand, epithelial-to-mesenchymal transition, TNF-α signaling through NFκB, and MYC targets v2 also reached statistical significance (Supplementary Table 13). Of these, the HALLMARK EPITHELIAL MESENCHYMAL TRANSITION gene set was also enriched in the 1 vs 0 nM DHT comparison in the LNCaP-ARhi cells, consistent with their higher androgen sensitivity (Supplementary Table 14). Collectively, these results highlight the difference between AR-expressing cell clones of normal epithelial and cancerous origin.

### Discussion

In this study, we established a cell model based on the nontransformed human papilloma virus 18 immortalized prostate epithelial cell line RWPE-1 (Bello *et al.* 1997), by introducing AR expression to the cells. The parental RWPE-1 cells were originally reported to be AR positive (Bello *et al.* 1997), but our results at both the mRNA and protein levels, as well as results from others, indicate that the cells have lost expression of AR during culture (Prensner *et al.* 2011, Fang *et al.* 2013). Here, AR signaling was partially rescued in RWPE-1-AR cells, as shown by the expression of AR target genes, such as *FKBP5*, *ZBTB16,* and to a certain extent *TMPRSS2,* following DHT stimulation. The induction of certain notable AR target genes, such as *KLK3* and *NKX3-1*, was not detected, indicating that either a gene regulatory factor or an external signal that induces expression of these genes was missing.

RWPE-1-AR cells express luminal cytokeratins (*KRT8* and *KRT18*) but also lower levels of basal markers (*KRT5, KRT14,* and *TP63*). Expression of both luminal and basal cytokeratins has been previously associated with transit-amplifying cells, a cell type that is differentiating from basal stem cells into more differentiated cells of the prostate lumen. Bipotential stem cells, such as transit-amplifying cells, are often considered to be the cell population that gives rise to carcinomas, including prostate adenocarcinoma (Wang *et al.* 2009, Agarwal *et al.* 2015, Guo *et al.* 2020). Interestingly, we found that *KRT5* was induced in RWPE-1-AR cells in response to DHT. *KRT5* was previously identified as an AR target gene in HPr-1 AR cells by Bolton *et al.* (2007). However, the expression levels of luminal marker genes, especially in RWPE-1-AR cells, are 10-fold higher than those of basal markers showing that the cells lean toward luminal differentiation.

In addition to transit-amplifying cell markers, RWPE-1 cells also express markers of club cells and hillock cells and those of related luminal progenitor cells (Henry *et al.* 2018, Guo *et al.* 2020, Karthaus *et al.* 2020). Interestingly, Guo *et al.* (2020) showed that differentiated luminal cells have delayed tumor onset when transformed in comparison to luminal progenitor cells, potentially due to their having to dedifferentiate to progenitor cells to gain proliferative capacity (Guo *et al.* 2020). This suggests that RWPE-1-AR cells can serve as a proper model system to study the transformation process in human cells.

Androgen signaling has a dual role in the normal prostate. The autocrine effect of androgens in the prostate epithelium is differentiation instead of growth. However, androgens also have paracrine actions in the prostate, leading to the growth stimulation of the prostate epithelium. In the early development of PCa, there is a shift in autocrine androgen action from differentiation to proliferation (Gao *et al.* 2001). Consistent with this, we observed significant growth reduction caused by DHT in AR-expressing RWPE-1 cells, suggesting that RWPE-1-AR cells recapitulate the autocrine action of androgens in normal prostate epithelial cells.

Cell growth can be affected by both proliferation and death. Cell cycle arrest, apoptosis, or both may account for some of the observed reduction on growth, but neither was found to be a statistically significant culprit. Our mRNA sequencing results indicated a reduction in the activity of the mitogenic KRAS pathway. We documented KRAS-pathway suppression in RWPE-1-AR cells following short-term (4 h) exposure to androgens. The reduction of KRAS activity was further validated by the reduction of MAPK ERK1/2 activation at 24 h in both AR-expressing RWPE-1 clones and in ARc15 clone also after 48 h DHT exposure. Thus, the KRAS pathway plays a role in the growth reduction of RWPE-1-AR cells upon DHT stimulation. Previously, it was shown that androgens lead to growth reduction in hTERT-immortalized PrEC-AR cells via downregulation of myc proto-oncogene protein encoded by *MYC* (Ling *et al.* 2001, Antony *et al.* 2014). In our model, MYC signaling was significantly downregulated at the unstimulated condition in RWPE-1-AR cells compared to controls. Although *MYC* expression in RWPE-1-AR cells was slightly reduced after DHT stimulation, the reduction was not statistically significant in any comparison nor did the MYC target gene sets become enriched.

We also detected small but significant increase in the cell motility following restoration of AR signaling in the RWPE-1 cells. Cell migration is a complex process involving many proteins of the cytoskeleton, including carefully orchestrated assembly of actin filaments to form protrusions such as filopodia and lamellipodia to direct the movement, and activation of integrins to attach to the matrix. Previously, it has been shown in mouse fibroblasts that following androgen stimulus, AR forms a complex with filamin A and activates integrin β1 leading to a cascade that increases cellular movement (Castoria *et al.* 2011). Whether similar mechanism is activated in prostate epithelial cells and is responsible for the seen effect in RWPE-1-AR cell motility remains to be studied.

The androgen response of RWPE-1-AR cells is markedly different from that of the cancer cell lines LNCaP-pcDNA3.1 and -ARhi, as shown by the differences in the growth response as well as in the induction of genes following DHT stimulation. In LNCaP cells, growth signaling is induced by androgens, and AR activates the epithelial-to-mesenchymal transition signature as well as NFκB signaling. In contrast, androgen treatment leads to suppression of NFκB and KRAS signaling in RWPE-1-AR cells. These differences in growth signaling are accompanied by the enrichment of cancer-related AR signatures in LNCaP cells (Pomerantz *et al.* 2015) and normal-related AR signatures in RWPE-1-AR cells.

In summary, we presented a novel prostate epithelial cell line model expressing AR. Based on the normal prostate-like gene expression pattern, progenitor-like characteristics, and androgen responsiveness, these RWPE-1-AR cells can be utilized in the future to study the early tumorigenesis of the prostate. Further modification of the model allows dissection of the effects of different PCa driver events alone or in combination.

## Supplementary Material

Supplementary Figure 1

Supplementary Table 1. RWPE-1-AR and Ctrl marker genes. Overlap of top 100 DE genes in RWPE-1-ARc5 vs RWPE-1-Ctrlc1 at 0 nM DHT and RWPE-1-ARc15 vs RWPE-1-Ctrlc1 at 0 nM DHT comparisons are shown. Details of the differential expression analysis results are shown first for ARc5 vs Ctrlc1 and then for ARc15 vs Ctrlc1.

Supplementary Table 2. RWPE-1-AR and Ctrl marker genes at 1 nM DHT. Overlap of top 100 DE genes in RWPE-1-ARc5 vs RWPE-1-Ctrlc1 at 1 nM DHT and RWPE-1-ARc15 vs RWPE-1-Ctrlc1 at 1 nM DHT comparisons are shown. Details of the differential expression analysis results are shown first for ARc5 vs Ctrlc1 and then for ARc15 vs Ctrlc1.

Supplementary table 3. RWPE-1-AR and Ctrl marker genes. Overlap of top 100 DE genes in RWPE-1-ARc5 vs RWPE-1-Ctrlc1 at 100 nM DHT and RWPE-1-ARc15 vs RWPE-1-Ctrlc1 at 100 nM DHT comparisons are shown. Details of the differential expression analysis results are shown first for ARc5 vs Ctrlc1 and then for ARc15 vs Ctrlc1.

Supplementary table 4. List of mutually upregulated genes in RWPE-1-AR clones and LNCaP-pcDNA3.1 in 100 vs 0 nM DHT.

Supplementary table 5. List of mutually upregulated genes in RWPE-1-AR clones and LNCaP-ARhi in 100 vs 0 nM DHT.

Supplementary table 6. List of mutually downregulated genes in RWPE-1-AR clones and LNCaP-ARhi in 100 vs 0 nM DHT.

Supplementary table 7. Significantly enriched gene sets in 1 nM DHT vs 0 nM DHT in RWPE-1-ARc5.

Supplementary table 8. Significantly enriched gene sets in 100 nM DHT vs 0 nM DHT in RWPE-1-ARc5.

Supplementary table 9. Significantly enriched genesets in 1 nM vs 0 nm DHT in RWPE-1-ARc15.

Supplementary table 10. Significantly enriched gene sets in 100 nM vs 0 nm DHT in RWPE-1-ARc15.

Supplementary table 11. Comparison of RWPE-1-ARc5 and ARc15 cells against RWPE-1-Ctrlc1 in 0 nM DHT.

Supplementary table 12. Significantly enriched gene sets in 100 nM vs 0 nM DHT in LNCaP-pcDNA3.1

Supplementary table 13. Significantly enriched genes sets in 100 vs 0 nM DHT in LNCaP-ARhi.

Supplementary table 14. Significantly enriched gene sets in 1 nM vs 0 nM DHT in LNCaP-ARhi.

Supplementary table 15. List of primary antibodies.

Supplementary table 16. List of primers.

## Conflicts of interest

None of the authors have nothing to declare

## Funding

This work was supported by the Academy of Finland (LL 317871, 334774; MN 312043; TV 317755), Sigrid Jusélius Foundation (LL, MN, TV), Cancer Foundation Finland (LL, MN), the Competitive State Research Financing of the Expert Responsibility area of Tampere University Hospital (TV), The Finnish Cultural Foundation (KK), Tampere University, Faculty of Medicine and Health Technology (BK).
