# Supplementary material for: Nonmalignant AR-positive prostate epithelial cells and cancer cells respond differently to androgen
Source: Endocr Relat Cancer. 2022 Oct 10;29(12):717–33. doi: 10.1530/ERC-22-0108 (PMC9644224; doi:10.1530/ERC-22-0108)
Supplement: Supplementary Figure 1 [file supplementary_figure_1.pdf]

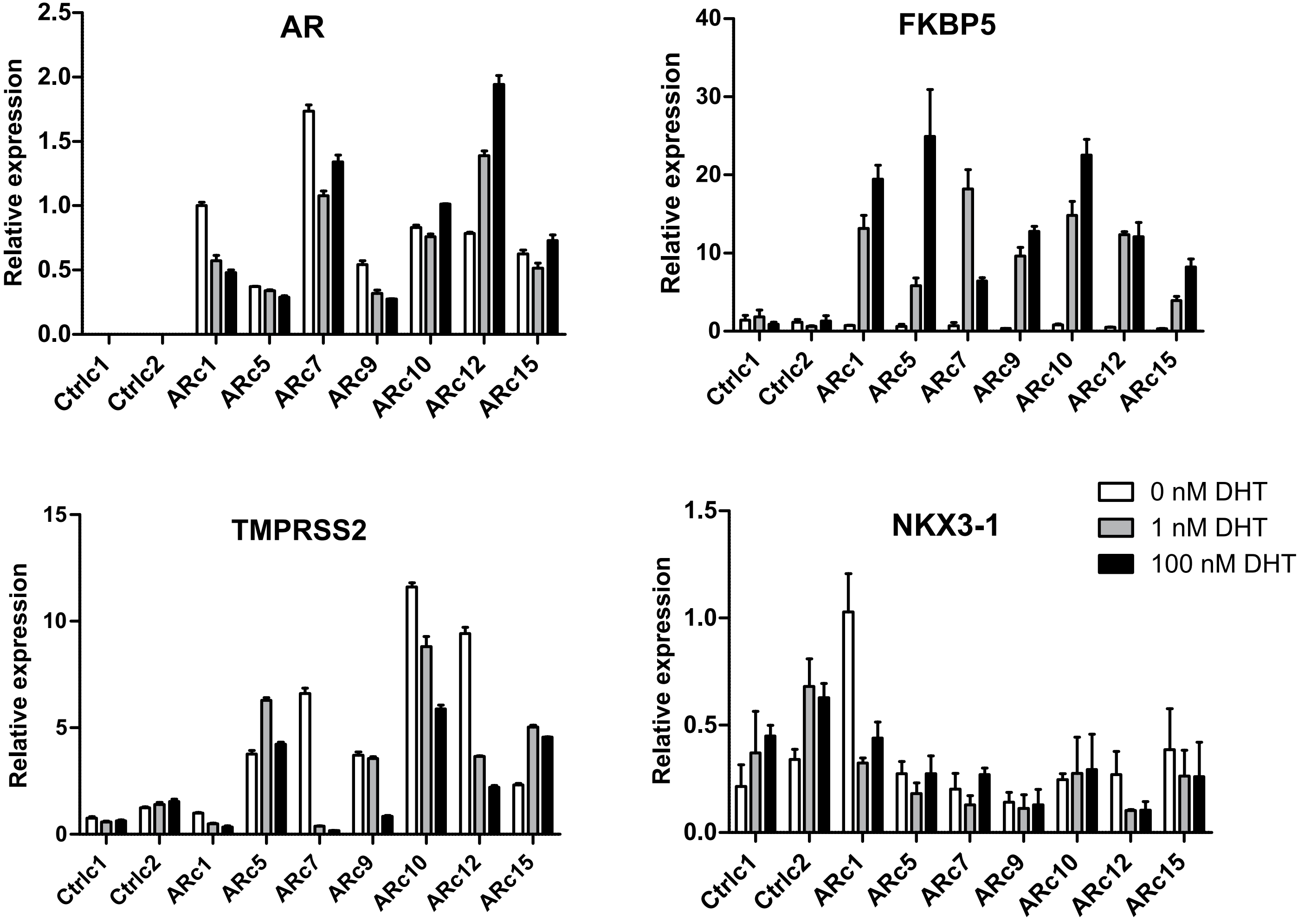

Supplementary Figure 1. The initial screening of RWPE-1-AR clones. AR and its target genes FKBP5, TMPRSS2 and NKX3-1 were quantified in the mRNA level using RT–qPCR after 24 h stimulation with indicated concentrations of DHT. Bars represent mean and S.E.M of 3 technical replicates.

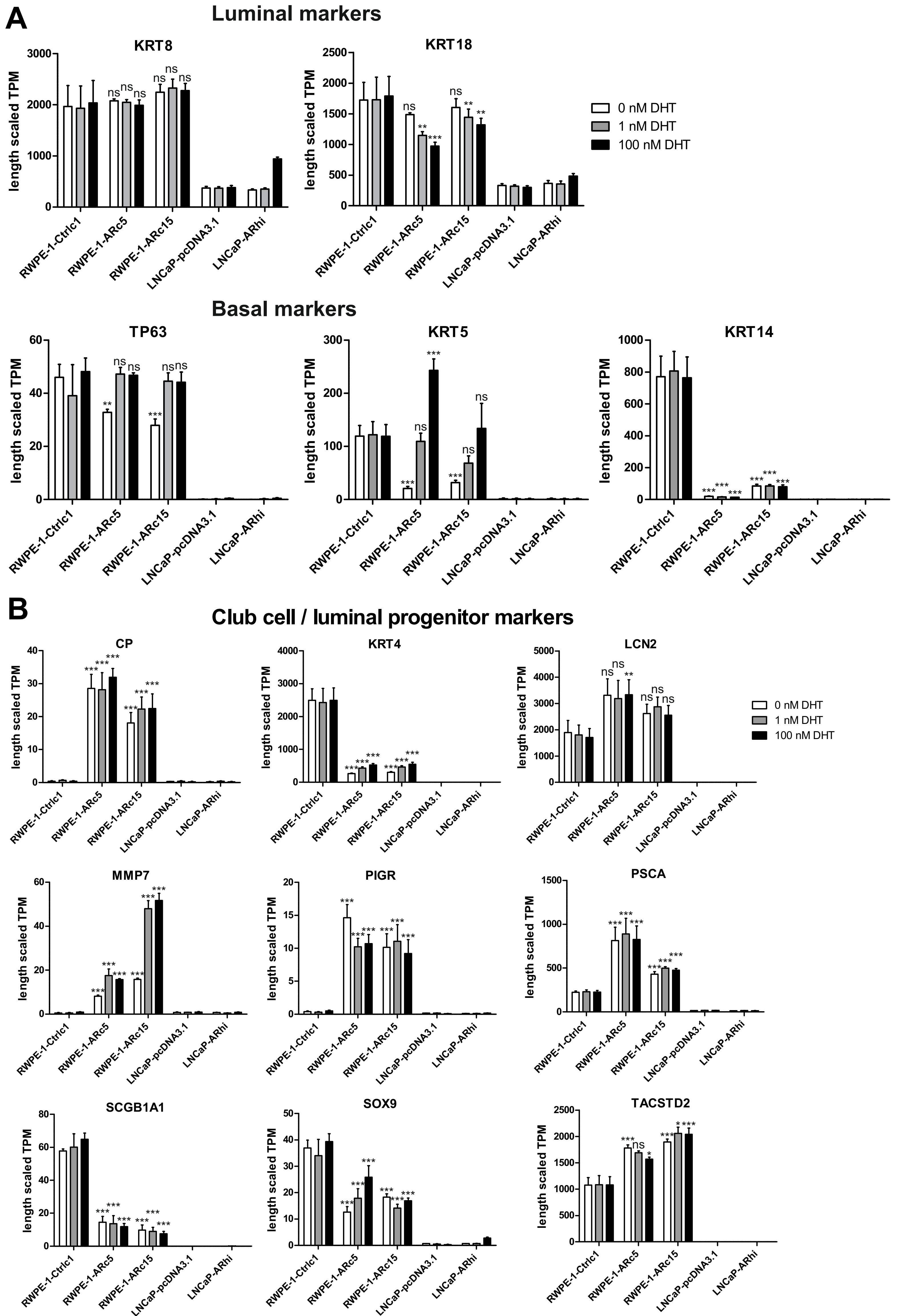
A
Luminal markers
KRT8
KRT18
Basal markers
TP63
KRT5
KRT14
B
Club cell / luminal progenitor markers
CP
KRT4
LCN2
MMP7
PIGR
PSCA
SCGB1A1
SOX9
TACSTD2
length scaled TPM
0 nM DHT
1 nM DHT
100 nM DHT
RWPE-1-Ctrlc1
RWPE-1-ARc5
RWPE-1-ARc15
LNCaP-pcDNA3.1
LNCaP-ARhi

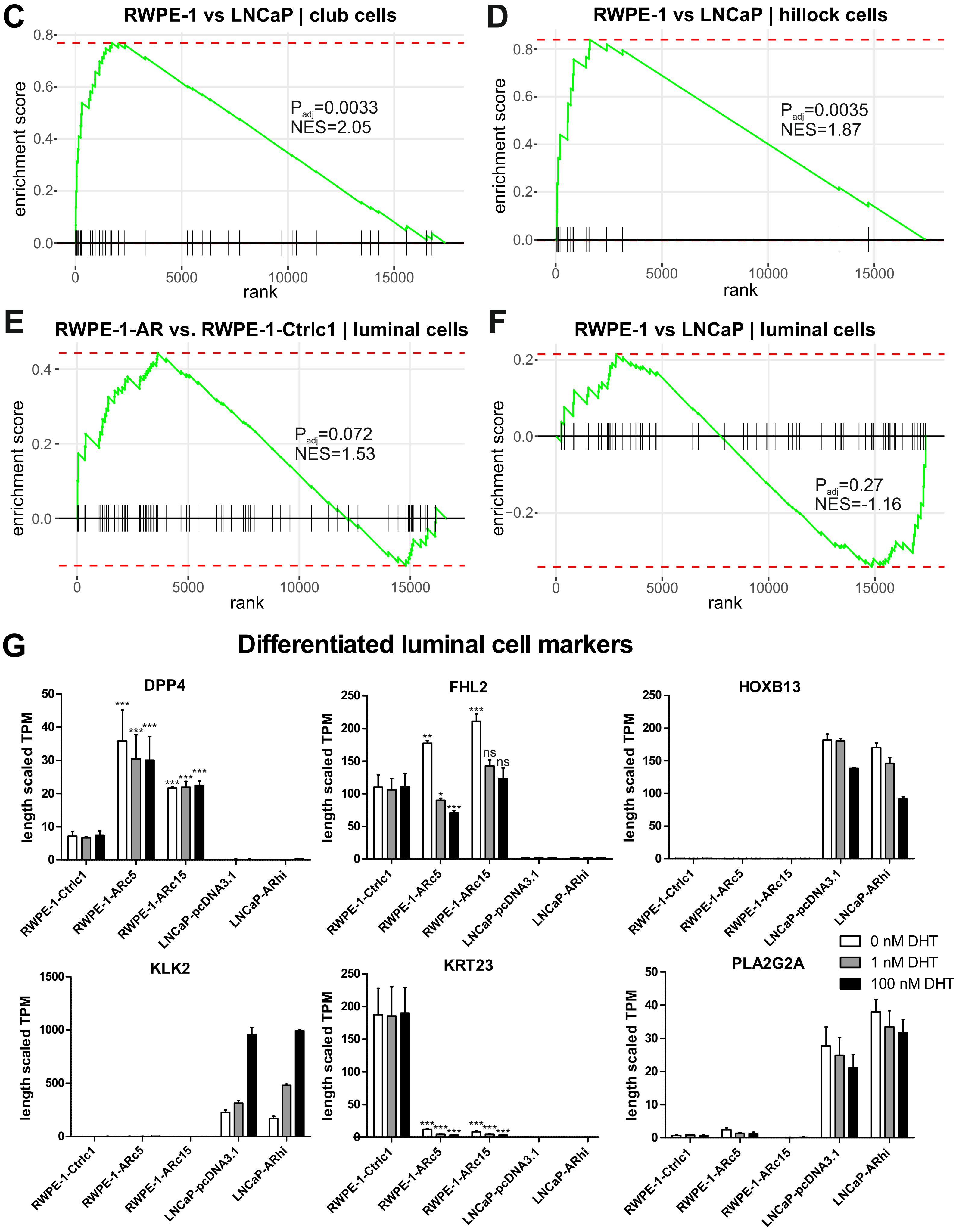

Supplementary Figure 2. A) and B) The expression of markers for canonical prostate luminal and basal epithelial cells and the luminal progenitor/club cells across RWPE-1 and LNCaP samples. B–H adjusted p values from Wald test for gene expression in comparison to Ctrlc1 are indicated for AR expressing RWPE-1 clones with * < 0.05, ** < 0.01, and *** < 0.001, ns not significant. The human club and luminal progenitor cell markers from Henry et al. 2018 (PSCA, PIGR, MMP7, LCN2), Karthaus et al. 2020 (PSCA, KRT4, SCGB1A1), and Guo et al. 2020 (all). C) GSEA result of the differentiated club cell signature between RWPE-1-AR and control cells (all samples). D) GSEA result of the hillock cell signature between RWPE-1-AR and control cells (all samples). E) GSEA result of the differentiated luminal cell signature between RWPE-1-AR and control cells (all samples). F) GSEA result of the differentiated luminal cell signature between RWPE-1 (all samples) and LNCaP (all samples). G) The expression of markers for differentiated luminal cells from Henry et al. 2018 (PLA2G2A, KLK2), Karthaus et al. 2020 (DPP4, PLA2G2A) as well as from Guo et al. 2020 (HOXB13, KLK2, FHL2, and KRT23). KLK3 and NKX3-1 shown in Figure 2A are also markers for differentiated luminal cells.

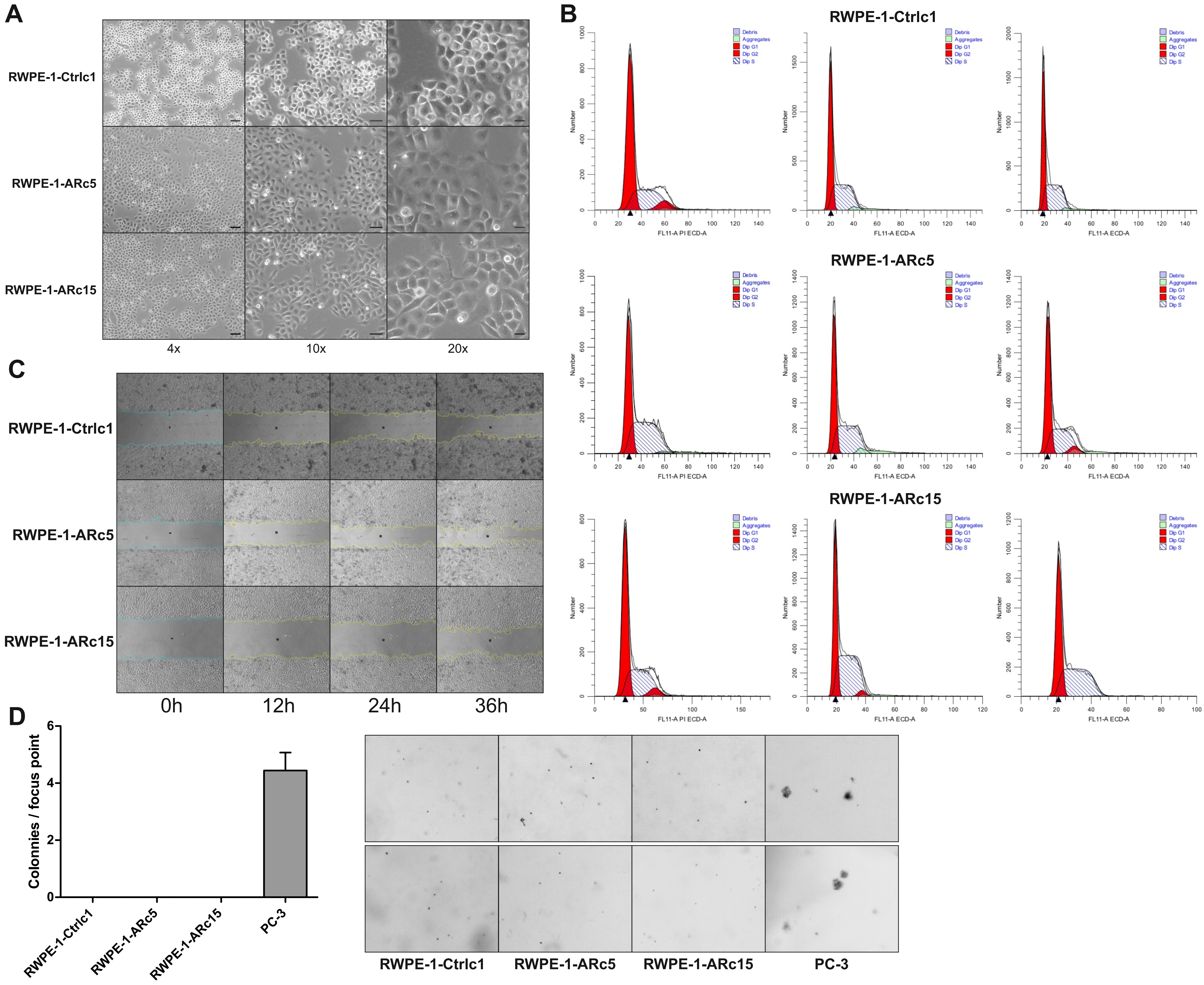

Supplementary Figure 3. A) The morphology of RWPE-1-AR and control clones in K-SFM 72 h after seeding. Scalebars 100 µm, 50 µm and 20 µm, for 4x, 10x and 20x, respectively. B) Histrograms of cell cycle data measured for the PI channel. Cells in G1, S and G2 phase are modelled as well as debris and aggregated cells. C) An image panel representative of the wound healing assay. D) Colony formation assay. Cells were seeded in to 3 wells / cell line. After 3 weeks, 3 focus points were randomly selected from each well and imaged with 4x magnification and colonies were counted. Left, the quantitation result from the first assay. Right, an image panel representative of the colony formation assay. Images shown are from two experiments with similar results.

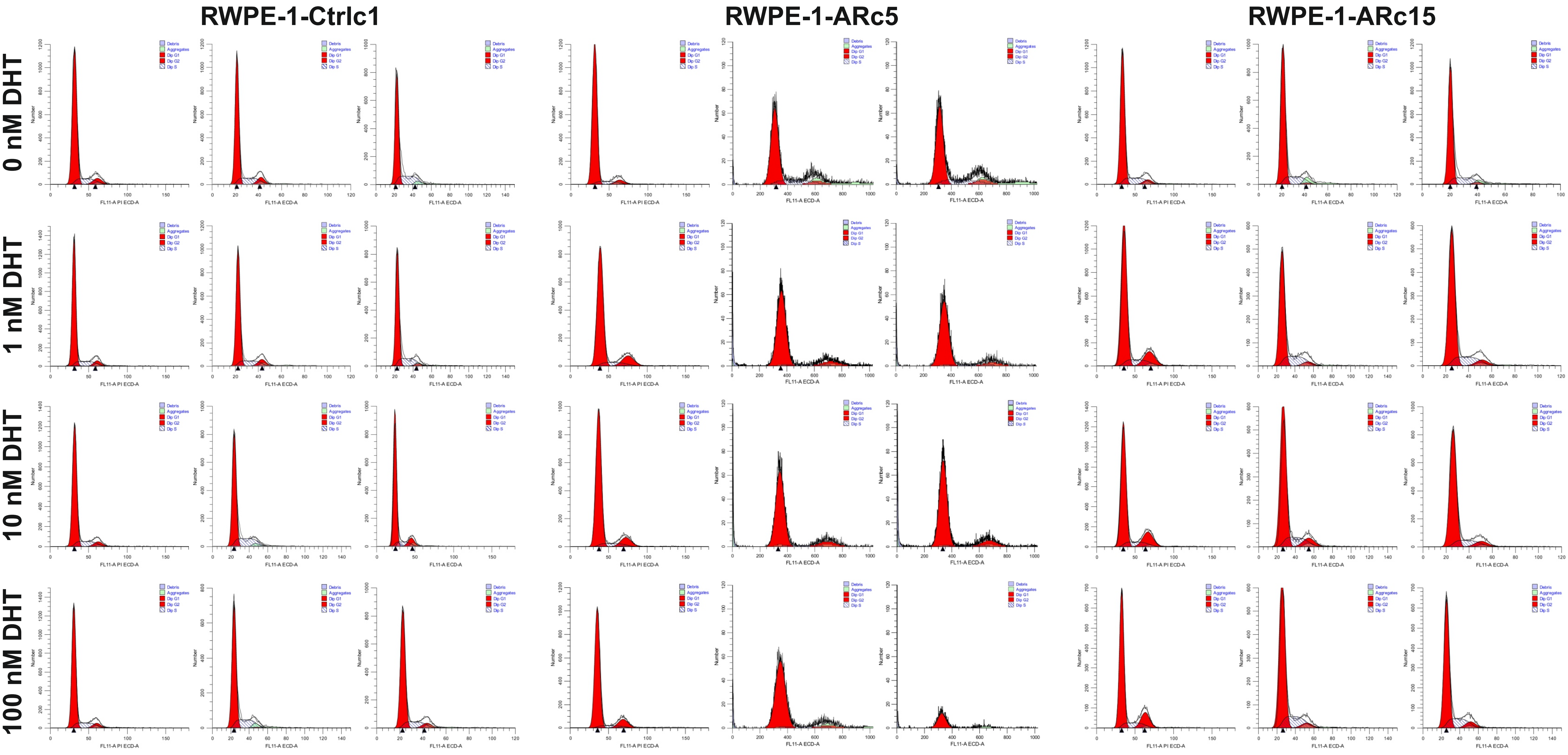

Supplementary figure 4. Cell cycle analysis. Histograms of the cell cycle analysis data for the DHT treated RWPE-1-AR and control cells. Cells in G1, S and G2/M phase are modelled as is debris and aggregates.

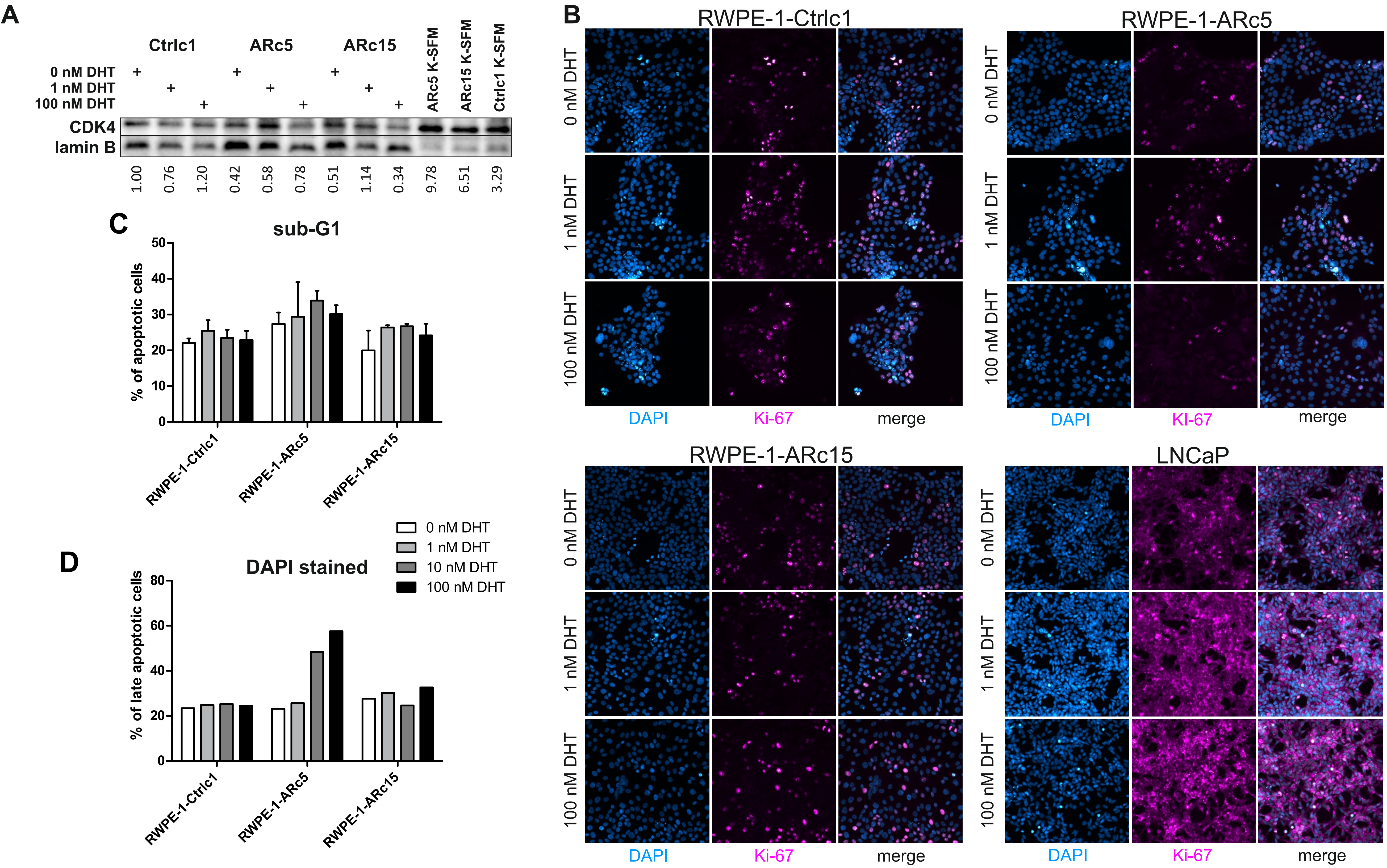

Supplementary figure 5. Effect of DHT to actively proliferating and apoptotic cells. A) CDK4 expression in RWPE-1-AR and control cells was measured by Western blot across different DHT stimulated conditions, as well as in the native K-SFM cell culture medium. Ki-67 staining of RWPE-1-Ctrlc1, -ARc5, -ARc15, and LNCaP (parental) as positive control. B) The percentages of PI-stained permeabilized cells with DNA content sub-G0/G1 peak in the cell cycle analysis data. Mean and S.E.M of 3 replicates are shown. C) The percentages of nonpermeabilized DAPI-stained cells in the analysis for apoptosis using Annexin V staining in each condition as measured by flow cytometry. The percentage of Annexin V-stained cells was very low in all conditions so late apoptotic cells identified by staining with DAPI are shown.

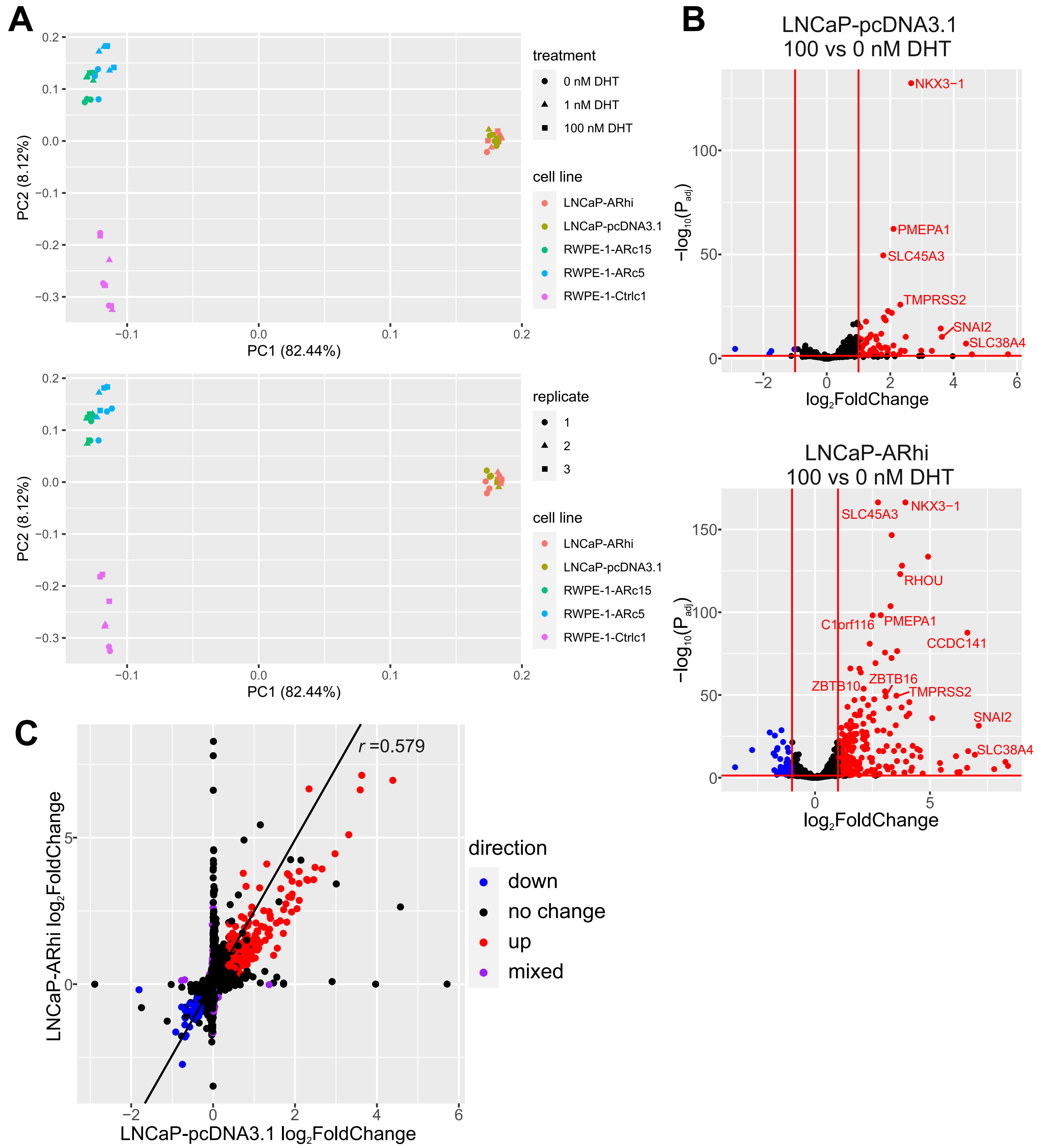

Supplementary Figure 6. A) A principal component analysis of the samples in RNA-seq. B) Differential expression following stimulation with 100 nM DHT in LNCaP-pcDNA3.1 and -ARhi cells. Significantly upregulated genes are shown in red and downregulated in blue. The horizontal line indicates the threshold of statistical significance (B–H adjusted $p < 0.05$) and the vertical lines log2fc of -1 and 1. Some genes mutually regulated in both cell lines are labelled. C) Correlation of the log2fc of the two LNCaP cell lines following stimulation with 100 nM DHT. Genes statistically significantly upregulated (red) and downregulated (blue) in both cell lines. Genes with mixed (purple) expression change (statistically significant up- or downregulation in one cell line and other direction of change in the other cell line).

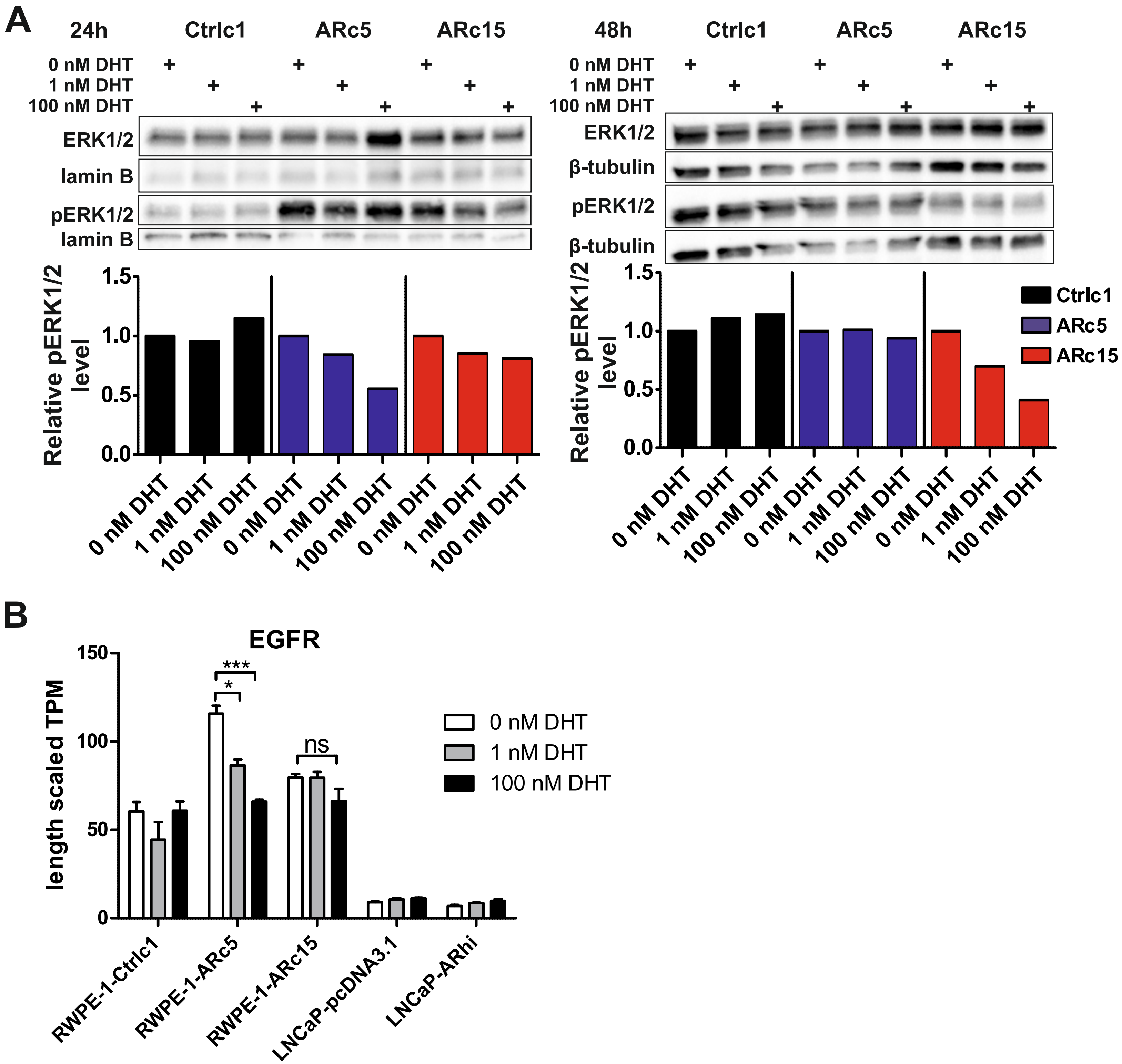

Supplementary figure 7. A) KRAS pathway activation was studied by measuring the amount of active ERK1/2 MAP kinase in different levels of DHT using Western blot after 24h and 48h of DHT stimulation. Each protein was first quantitated relative to loading control and then pERK1/2 relative to total ERK1/2. The amount of pERK1/2 at 0 nM DHT sample was then set to 1 for each cell line. B) Expression of KRAS signaling pathway upstream effector, EGFR was studied from the mRNA-sequencing data. Mean and S.E.M. of 3 replicates are shown. * $P < 0.05$, *** $P < 0.001$.
