# Supplementary material for: Nonmalignant AR-positive prostate epithelial cells and cancer cells respond differently to androgen
Source: Endocr Relat Cancer. 2022 Oct 10;29(12):717–33. doi: 10.1530/ERC-22-0108 (PMC9644224; doi:10.1530/ERC-22-0108)
Supplement: Supplementary Table 1. RWPE-1-AR and Ctrl marker genes. Overlap of top 100 DE genes in RWPE-1-ARc5 vs RWPE-1-Ctrlc1 at 0 nM DHT and RWPE-1-ARc15 vs RWPE-1-Ctrlc1 at 0 nM DHT comparisons are shown. Details of the differential expression analysis results are shown first for ARc5 vs Ctrlc1 and then for [file supplementary_table_1.pdf]

Supplementary table 1. Supplementary Table 1. RWPE-1-AR and Ctrl marker genes. Overlap of top 100 DE genes in RWPE-1-ARc5 vs RWPE-1-Ctrlc1 at 0 nM DHT and RWPE-1-ARc15 vs RWPE-1-Ctrlc1 at 0 nM DHT comparisons are shown. Details of the differential expression analysis results are shown first for ARc5 vs Ctrlc1 and then for ARc15 vs Ctrlc1.

		ARc5 vs Ctrlc1 0 nM DHT					ARc15 vs Ctrlc1 0 nM DHT				
Ensemble gene id	Hgnc symbol	baseMean	log_2FC	lfcSE	P	P_{adj}	baseMean	log_2FC	lfcSE	P	P_{adj}
ENSG00000169083	AR	25906	10,74	0,18	0	0	14268	9,94	0,18	0	0
ENSG00000205420	KRT6A	8560	-6,79	0,20	6,5E-242	5,1E-238	8190	-6,12	0,42	6,63E-50	2,31E-47
ENSG00000164687	FABP5	17104	-8,10	0,31	2,7E-155	1,4E-151	16376	-7,61	0,39	1,81E-87	1,85E-84
ENSG00000133710	SPINK5	10753	-8,64	0,33	2,7E-154	1,1E-150	11532	-7,82	0,26	2,1E-197	1,6E-193
ENSG00000143556	S100A7	6475	-7,82	0,33	3,5E-123	1,1E-119	6158	-9,91	0,59	5,23E-64	2,86E-61
ENSG00000185479	KRT6B	1094	-5,63	0,25	8,9E-117	2,3E-113	1075	-4,23	0,25	2,18E-67	1,29E-64
ENSG00000186847	KRT14	10120	-5,36	0,24	1,1E-109	2,5E-106	10403	-3,21	0,24	9,28E-42	2,29E-39
ENSG00000124429	POF1B	1257	-7,57	0,37	4,33E-96	8,49E-93	1234	-3,40	0,24	5,68E-47	1,71E-44
ENSG00000167754	KLK5	2563	-5,52	0,28	4,68E-90	8,16E-87	2425	-5,96	0,25	9,5E-132	3,6E-128
ENSG00000016602	CLCA4	850	-5,32	0,27	3,99E-87	6,25E-84	810	-4,94	0,25	6,79E-88	7,43E-85
ENSG00000204385	SLC44A4	473	4,69	0,24	8,24E-83	1,17E-79	610	5,14	0,20	2,3E-152	1,2E-148
ENSG00000178934	LGALS7B	941	-7,87	0,43	1,03E-75	1,24E-72	901	-7,30	0,55	5,71E-41	1,37E-38
ENSG00000188089	PLA2G4E	692	-7,45	0,41	1,65E-72	1,85E-69	659	-7,06	0,38	1,55E-78	1,32E-75
ENSG00000047457	CP	630	5,97	0,34	4,02E-72	4,2E-69	409	5,38	0,36	4,41E-52	1,73E-49
ENSG00000124466	LYPD3	4742	-3,97	0,23	6,87E-70	6,73E-67	4641	-3,43	0,18	1,9E-81	1,71E-78
ENSG00000169469	SPRR1B	5670	-9,70	0,56	1,21E-68	1,11E-65	5413	-9,46	0,48	1,31E-88	1,54E-85
ENSG00000106278	PTPRZ1	747	-8,36	0,48	2,48E-67	2,16E-64	762	-6,91	0,33	8,98E-98	1,53E-94
ENSG00000186395	KRT10	16725	-3,03	0,18	7,4E-67	6,1E-64	15685	-2,77	0,14	6,27E-91	8,01E-88
ENSG00000186832	KRT16	4987	-4,50	0,26	2,56E-66	2,01E-63	5163	-2,93	0,24	1,01E-34	1,77E-32
ENSG00000198691	ABCA4	447	-4,66	0,28	1,63E-65	1,21E-62	451	-5,59	0,30	7,91E-77	6,38E-74
ENSG00000131089	ARHGEF9	374	-3,69	0,22	4,26E-65	3,03E-62	343	-4,35	0,21	4,74E-97	7,26E-94
ENSG00000105388	CEACAM5	698	-7,75	0,47	1,02E-60	6,68E-58	729	-7,27	0,62	2,93E-33	4,78E-31
ENSG00000198483	ANKRD35	334	-5,97	0,37	1,85E-60	1,16E-57	325	-5,31	0,31	7,49E-68	4,99E-65
ENSG00000143546	S100A8	20705	-8,92	0,55	5,86E-60	3,53E-57	19711	-10,49	0,78	1,1E-42	2,81E-40
ENSG00000121552	CSTA	4489	-5,36	0,33	8,07E-60	4,68E-57	4240	-5,44	0,47	8,56E-32	1,33E-29
ENSG00000142973	CYP4B1	555	-3,42	0,22	2,23E-57	1,25E-54	536	-5,20	0,23	5,3E-111	1,6E-107
ENSG00000126947	ARMCX1	210	3,99	0,25	4,43E-57	2,4E-54	167	3,72	0,25	2,21E-51	8,28E-49
ENSG00000169509	CRCT1	773	-5,61	0,36	1,08E-54	5,47E-52	754	-4,53	0,33	9,2E-45	2,56E-42
ENSG00000162896	PIGR	492	5,05	0,33	3,98E-54	1,95E-51	343	4,57	0,35	1,17E-39	2,61E-37
ENSG00000179477	ALOX12B	254	-6,21	0,41	2,26E-53	1,07E-50	255	-5,79	0,36	2,32E-59	1,11E-56

ENSG00000129437	KLK14	390	-4,61	0,31	4,34E-52	1,94E-49	371	-4,83	0,30	1,42E-57	6,04E-55
ENSG00000125730	C3	33682	3,49	0,23	3,55E-51	1,55E-48	22720	2,94	0,23	3,14E-37	6,26E-35
ENSG00000101144	BMP7	501	-8,08	0,55	1,81E-49	7,48E-47	505	-8,06	0,54	5,41E-51	1,97E-48
ENSG00000170477	KRT4	45439	-3,36	0,23	9,19E-49	3,6E-46	44024	-3,07	0,21	5,23E-49	1,71E-46
ENSG00000166535	A2ML1	1031	-4,63	0,32	9,83E-48	3,67E-45	1009	-5,36	0,31	7,48E-70	5,46E-67
ENSG00000143631	FLG	515	-4,21	0,30	2,16E-45	7,2E-43	475	-5,57	0,32	8,12E-68	5,19E-65
ENSG00000106714	CNTNAP3	1402	3,35	0,24	5,49E-45	1,76E-42	851	2,68	0,13	1,9E-102	4,1E-99
ENSG00000143369	ECM1	448	-4,38	0,32	1,97E-44	6,19E-42	431	-3,73	0,32	2,19E-32	3,46E-30
ENSG00000118402	ELOVL4	605	-3,86	0,28	6,2E-44	1,9E-41	613	-2,77	0,23	5,41E-34	9,11E-32
ENSG00000181458	TMEM45A	407	-4,85	0,36	2,98E-43	8,8E-41	361	-5,29	0,37	1,48E-47	4,53E-45
ENSG00000081277	PKP1	21852	-2,11	0,16	3,77E-41	1,07E-38	22225	-1,67	0,12	2,84E-43	7,5E-41
ENSG00000167768	KRT1	6188	-10,12	0,77	6,9E-41	1,93E-38	5870	-13,22	0,98	1,78E-40	4,14E-38
ENSG00000000971	CFH	194	3,45	0,27	2,34E-39	6,22E-37	401	4,63	0,29	4,68E-60	2,32E-57
ENSG00000284981	UPK3BL2	2197	-2,34	0,19	1,15E-37	2,86E-35	2170	-2,07	0,15	4,69E-43	1,22E-40
ENSG00000134757	DSG3	2712	-2,04	0,16	1,72E-37	4,21E-35	2784	-1,52	0,12	1,13E-37	2,27E-35
ENSG00000140945	CDH13	372	-2,45	0,20	7,49E-37	1,75E-34	334	-3,35	0,27	7,94E-38	1,64E-35
ENSG00000134755	DSC2	2159	-1,95	0,16	1,47E-36	3,34E-34	2065	-1,88	0,14	6,18E-40	1,41E-37
ENSG00000154529	CNTNAP3B	1699	2,50	0,20	3,83E-36	8,12E-34	1495	2,50	0,12	1,4E-104	3,6E-101
ENSG00000174501	ANKRD36C	971	2,97	0,24	3,97E-36	8,3E-34	688	2,53	0,19	7,35E-40	1,66E-37
ENSG00000108582	CPD	2618	1,61	0,13	6,34E-36	1,29E-33	2240	1,39	0,11	6,98E-37	1,36E-34
ENSG00000215853	RPTN	484	-4,04	0,33	1,18E-35	2,38E-33	446	-5,26	0,33	6,4E-58	2,81E-55
ENSG00000267368	UPK3BL1	6177	-2,64	0,22	1,39E-35	2,76E-33	5989	-2,49	0,16	1,02E-58	4,73E-56
ENSG00000179059	ZFP42	163	-3,86	0,32	1,47E-35	2,87E-33	158	-3,59	0,30	2,01E-34	3,43E-32
ENSG00000121742	GJB6	890	-3,41	0,28	2,65E-34	5,07E-32	786	-4,19	0,27	5,14E-54	2,13E-51
ENSG00000137975	CLCA2	485	-4,52	0,38	1,39E-33	2,58E-31	453	-5,06	0,36	4,91E-45	1,39E-42
ENSG00000117114	ADGRL2	527	-3,55	0,30	3,05E-33	5,5E-31	492	-3,39	0,27	7,95E-37	1,52E-34
ENSG00000163406	SLC15A2	665	3,46	0,30	1,68E-31	2,86E-29	760	3,81	0,22	9,8E-68	6,01E-65
