# Supplementary material for: Nonmalignant AR-positive prostate epithelial cells and cancer cells respond differently to androgen
Source: Endocr Relat Cancer. 2022 Oct 10;29(12):717–33. doi: 10.1530/ERC-22-0108 (PMC9644224; doi:10.1530/ERC-22-0108)
Supplement: Supplementary Table 2. RWPE-1-AR and Ctrl marker genes at 1 nM DHT. Overlap of top 100 DE genes in RWPE-1-ARc5 vs RWPE-1-Ctrlc1 at 1 nM DHT and RWPE-1-ARc15 vs RWPE-1-Ctrlc1 at 1 nM DHT comparisons are shown. Details of the differential expression analysis results are shown first for ARc5 vs Ctrlc1  [file supplementary_table_2.pdf]

Supplementary table 2. Supplementary Table 2. RWPE-1-AR and Ctrl marker genes at 1 nM DHT. Overlap of top 100 DE genes in RWPE-1-ARc5 vs RWPE-1-Ctrlc1 at 1 nM DHT and RWPE-1-ARc15 vs RWPE-1-Ctrlc1 at 1 nM DHT comparisons are shown. Details of the differential expression analysis results are shown first for ARc5 vs Ctrlc1 and then for ARc15 vs Ctrlc1.

		ARc5 vs Ctrlc1 1 nM DHT					ARc15 vs Ctrlc1 1 nM DHT				
Ensemble gene id	Hgnc symbol	baseMean	log_2FC	lfcSE	P	P_{adj}	baseMean	log_2FC	lfcSE	P	P_{adj}
ENSG00000169083	AR	23603	10,53	0,22	0	0	13929	9,81	0,21	0	0
ENSG00000164687	FABP5	18300	-7,64	0,22	5,3E-254	4,1E-250	18054	-7,09	0,23	8E-205	6,2E-201
ENSG00000143546	S100A8	22248	-9,29	0,30	6,4E-211	3,3E-207	22317	-10,53	0,37	4,7E-175	1,8E-171
ENSG00000169469	SPRR1B	6084	-10,08	0,35	2,7E-184	1E-180	6016	-10,24	0,36	6,7E-180	3,5E-176
ENSG00000186847	KRT14	10912	-5,93	0,22	1,7E-160	5,4E-157	11480	-3,44	0,21	2,76E-60	1,43E-57
ENSG00000205420	KRT6A	8793	-5,65	0,22	2E-142	5,2E-139	8651	-5,32	0,29	4,23E-76	4,38E-73
ENSG00000167754	KLK5	2796	-6,18	0,26	3,2E-129	7,2E-126	2729	-6,47	0,24	5,2E-165	1,6E-161
ENSG00000143556	S100A7	7137	-7,79	0,35	7,3E-113	1,4E-109	7128	-9,46	0,42	1,6E-115	3,6E-112
ENSG00000133710	SPINK5	12635	-8,14	0,36	5,3E-112	9,2E-109	12124	-6,96	0,34	3,46E-94	4,88E-91
ENSG00000185479	KRT6B	1137	-6,32	0,29	2,4E-109	3,8E-106	1146	-4,55	0,27	4,02E-64	2,61E-61
ENSG00000175040	CHST2	2629	5,66	0,26	5E-109	7,1E-106	1096	4,39	0,23	5,35E-84	5,93E-81
ENSG00000121552	CSTA	4784	-5,59	0,26	1,6E-103	2,1E-100	4737	-5,55	0,27	1,83E-95	2,85E-92
ENSG00000169429	CXCL8	891	-6,03	0,28	4,9E-103	5,9E-100	920	-5,01	0,22	1,3E-111	2,6E-108
ENSG00000250722	SELENOP	1932	6,03	0,28	6,5E-101	7,27E-98	497	4,02	0,28	1,17E-47	3,57E-45
ENSG00000144063	MALL	2134	-4,13	0,20	1,27E-96	1,32E-93	2431	-1,92	0,13	1,58E-47	4,71E-45
ENSG00000186395	KRT10	16680	-3,11	0,15	3,02E-92	2,94E-89	16537	-3,08	0,13	5E-125	1,3E-121
ENSG00000102760	RGCC	939	5,62	0,28	1,72E-91	1,57E-88	403	4,37	0,28	6,91E-55	2,62E-52
ENSG00000155893	PXYLP1	1430	3,69	0,19	1,91E-88	1,65E-85	889	2,94	0,18	1,08E-58	5,07E-56
ENSG00000144476	ACKR3	1939	4,03	0,20	3,01E-88	2,47E-85	1200	3,32	0,20	4,39E-66	3,25E-63
ENSG00000115112	TFCP2L1	4251	3,92	0,20	3,52E-83	2,74E-80	2373	3,03	0,23	1,1E-41	2,52E-39
ENSG00000108244	KRT23	2939	-5,51	0,30	5,45E-77	3,86E-74	2796	-5,42	0,31	5,04E-71	4,35E-68
ENSG00000267368	UPK3BL1	6359	-3,23	0,18	1,72E-71	1,17E-68	6371	-2,94	0,14	1,03E-95	1,77E-92
ENSG00000130052	STARD8	610	7,50	0,43	1,35E-67	8,74E-65	260	6,29	0,41	1,65E-52	5,68E-50
ENSG00000163207	IVL	16210	-3,41	0,20	3,57E-66	2,14E-63	17268	-2,35	0,15	3,07E-59	1,49E-56
ENSG00000016602	CLCA4	783	-5,64	0,34	1,03E-61	5,36E-59	788	-4,49	0,31	7,83E-48	2,43E-45
ENSG00000163216	SPRR2D	517	-7,84	0,47	3,86E-61	1,94E-58	512	-7,18	0,40	7,46E-71	6,1E-68
ENSG00000188089	PLA2G4E	622	-7,39	0,45	2,07E-60	1,01E-57	611	-6,66	0,42	3,56E-57	1,58E-54
ENSG00000106278	PTPRZ1	691	-7,63	0,48	1,25E-57	5,43E-55	685	-7,16	0,43	4,58E-64	2,84E-61
ENSG00000198691	ABCA4	442	-4,74	0,30	1,28E-56	5,12E-54	379	-5,50	0,37	7,34E-53	2,65E-50
ENSG00000179477	ALOX12B	288	-6,73	0,43	1,71E-56	6,67E-54	264	-6,08	0,40	1,64E-52	5,68E-50

ENSG00000163220	S100A9	30132	-4,35	0,28	2,12E-56	8,05E-54	29332	-5,01	0,32	9,8E-56	3,9E-53
ENSG00000169509	CRCT1	930	-5,44	0,35	5,98E-56	2,22E-53	922	-4,95	0,28	9,28E-72	8,48E-69
ENSG00000213022	KLK9	436	-6,79	0,43	6,77E-55	2,4E-52	542	-6,74	0,37	1,99E-73	1,93E-70
ENSG00000284981	UPK3BL2	2182	-2,74	0,18	8,74E-55	3,03E-52	2193	-2,49	0,16	3,57E-56	1,46E-53
ENSG00000106211	HSPB1	31189	-2,66	0,17	1,1E-54	3,74E-52	32430	-2,14	0,17	1,91E-37	3,19E-35
ENSG00000162896	PIGR	327	4,72	0,31	7,42E-54	2,41E-51	361	4,88	0,37	8,91E-41	1,92E-38
ENSG00000124466	LYPD3	5481	-2,87	0,19	3,7E-52	1,18E-49	5306	-3,03	0,18	2,39E-62	1,43E-59
ENSG00000105388	CEACAM5	635	-6,86	0,46	9,95E-51	3,04E-48	677	-6,83	0,49	1,09E-44	2,93E-42
ENSG00000008853	RHOBTB2	1827	2,73	0,18	1,08E-50	3,24E-48	1699	2,66	0,13	2,01E-90	2,6E-87
ENSG00000167656	LY6D	2119	-4,60	0,31	2,56E-50	7,4E-48	2250	-2,99	0,23	2,63E-40	5,46E-38
ENSG00000057149	SERPINB3	3196	-5,53	0,38	9,2E-50	2,61E-47	3178	-5,08	0,40	9,35E-38	1,58E-35
ENSG00000198483	ANKRD35	311	-6,42	0,44	6,58E-49	1,8E-46	319	-5,69	0,37	2,01E-53	7,45E-51
ENSG00000157404	KIT	175	5,13	0,35	2,84E-48	7,49E-46	120	4,59	0,36	3,01E-38	5,43E-36
ENSG00000101144	BMP7	530	-8,49	0,59	1,25E-47	3,19E-45	502	-7,99	0,53	2,23E-51	7,52E-49
ENSG00000241794	SPRR2A	339	-5,30	0,37	2,4E-47	5,94E-45	346	-4,02	0,31	4,08E-40	8,12E-38
ENSG00000172548	NIPAL4	402	-5,95	0,42	5,6E-47	1,36E-44	417	-4,22	0,35	1,81E-35	2,8E-33
ENSG00000125730	C3	28504	3,50	0,25	6,14E-46	1,41E-43	22662	3,17	0,25	3,48E-39	6,6E-37
ENSG00000215853	RPTN	421	-6,61	0,47	3,19E-45	7,1E-43	412	-7,14	0,55	7,4E-40	1,45E-37
ENSG00000131089	ARHGEF9	325	-3,28	0,24	5,54E-45	1,21E-42	279	-4,17	0,25	4,13E-62	2,38E-59
ENSG00000124102	PI3	53445	-3,58	0,26	2,54E-44	5,42E-42	52479	-3,74	0,26	3,79E-48	1,2E-45
ENSG00000184012	TMPRSS2	749	4,34	0,32	1,25E-43	2,53E-41	470	3,67	0,22	1,58E-64	1,06E-61
ENSG00000181458	TMEM45A	386	-5,94	0,43	1,71E-43	3,42E-41	400	-5,54	0,39	1,31E-46	3,83E-44
ENSG00000169474	SPRR1A	752	-9,41	0,68	2,4E-43	4,73E-41	767	-8,61	0,55	2,09E-55	8,13E-53
ENSG00000189334	S100A14	4909	-2,80	0,21	6,9E-43	1,34E-40	5115	-2,24	0,17	1,59E-42	3,7E-40
ENSG00000165474	GJB2	4006	-4,79	0,36	5,6E-42	1,06E-39	3977	-4,37	0,35	4,44E-37	7,18E-35
ENSG00000185483	ROR1	875	2,64	0,20	1,93E-41	3,58E-39	778	2,55	0,19	1,58E-42	3,7E-40
ENSG00000166828	SCNN1G	646	4,94	0,37	2,45E-41	4,49E-39	470	4,51	0,28	2,33E-61	1,29E-58
ENSG00000143147	GPR161	2975	1,89	0,14	4,61E-41	8,26E-39	2984	1,97	0,15	1,89E-39	3,63E-37
ENSG00000178372	CALML5	868	-4,53	0,35	2,24E-39	3,68E-37	852	-4,78	0,31	2,05E-56	8,6E-54
ENSG00000119899	SLC17A5	2628	2,57	0,20	1,24E-38	1,97E-36	2685	2,63	0,20	4,49E-41	9,83E-39
ENSG00000163406	SLC15A2	683	3,58	0,28	1,83E-38	2,89E-36	905	4,06	0,25	2,03E-59	1,02E-56
