# Supplementary material for: Nonmalignant AR-positive prostate epithelial cells and cancer cells respond differently to androgen
Source: Endocr Relat Cancer. 2022 Oct 10;29(12):717–33. doi: 10.1530/ERC-22-0108 (PMC9644224; doi:10.1530/ERC-22-0108)
Supplement: Supplementary table 3. RWPE-1-AR and Ctrl marker genes. Overlap of top 100 DE genes in RWPE-1-ARc5 vs RWPE-1-Ctrlc1 at 100 nM DHT and RWPE-1-ARc15 vs RWPE-1-Ctrlc1 at 100 nM DHT comparisons are shown. Details of the differential expression analysis results are shown first for ARc5 vs Ctrlc1 and then [file supplementary_table_3.pdf]

Supplementary table 3. RWPE-1-AR and Ctrl marker genes. Overlap of top 100 DE genes in RWPE-1-ARc5 vs RWPE-1-Ctrlc1 at 100 nM DHT and RWPE-1-ARc15 vs RWPE-1-Ctrlc1 at 100 nM DHT comparisons are shown. Details of the differential expression analysis results are shown first for ARc5 vs Ctrlc1 and then for ARc15 vs Ctrlc1.

		ARc5 vs Ctrlc1 100 nM DHT					ARc15 vs Ctrlc1 100 nM DHT				
Ensemble gene id	Hgnc symbol	baseMean	log_2FC	lfcSE	P	P_{adj}	baseMean	log_2FC	lfcSE	P	P_{adj}
ENSG00000169083	AR	23165	10,62	0,21	0,0E+00	0,0E+00	13925	9,92	0,19	0,0E+00	0,0E+00
ENSG00000096060	FKBP5	8902	3,30	0,10	6,0E-260	4,7E-256	5163	2,43	0,14	4,1E-72	2,0E-69
ENSG00000164687	FABP5	16152	-7,24	0,25	1,6E-183	8,2E-180	15910	-6,61	0,25	4,4E-158	3,4E-154
ENSG00000144063	MALL	1972	-4,73	0,16	4,9E-183	1,9E-179	2249	-2,22	0,15	8,2E-48	1,7E-45
ENSG00000133710	SPINK5	12561	-8,05	0,29	4,5E-173	1,4E-169	12351	-7,05	0,43	7,7E-62	2,8E-59
ENSG00000115112	TFCP2L1	5289	4,11	0,15	1,1E-165	2,8E-162	2994	3,25	0,17	1,2E-84	8,7E-82
ENSG00000185479	KRT6B	1018	-6,84	0,25	1,1E-160	2,3E-157	1029	-4,74	0,23	3,3E-97	4,6E-94
ENSG00000169469	SPRR1B	5605	-9,65	0,36	1,6E-157	3,1E-154	5549	-10,64	0,54	3,7E-87	3,0E-84
ENSG00000175040	CHST2	2851	6,15	0,23	4,1E-153	7,1E-150	1270	4,98	0,22	8,8E-116	1,9E-112
ENSG00000108551	RASD1	1479	7,96	0,30	5,4E-153	8,3E-150	298	5,61	0,42	2,6E-42	4,1E-40
ENSG00000155893	PXYLP1	1634	3,94	0,15	6,6E-152	9,3E-149	995	3,17	0,14	2,2E-110	3,8E-107
ENSG00000186847	KRT14	9369	-5,89	0,23	5,1E-146	6,6E-143	9965	-3,30	0,24	6,2E-44	1,1E-41
ENSG00000144476	ACKR3	2016	4,11	0,17	2,3E-136	2,7E-133	1314	3,48	0,18	1,5E-85	1,1E-82
ENSG00000250722	SELENOP	1809	6,15	0,25	6,2E-136	6,9E-133	558	4,42	0,24	5,5E-75	2,8E-72
ENSG00000205420	KRT6A	8127	-5,41	0,22	2,3E-131	2,2E-128	8017	-5,22	0,25	9,9E-98	1,5E-94
ENSG00000163207	IVL	14107	-3,92	0,16	8,3E-131	7,6E-128	15164	-2,61	0,15	7,0E-68	3,0E-65
ENSG00000167754	KLK5	2334	-6,18	0,26	6,0E-124	5,2E-121	2324	-6,32	0,24	9,7E-154	5,0E-150
ENSG00000102760	RGCC	967	5,79	0,25	6,0E-118	4,6E-115	472	4,75	0,27	3,4E-69	1,6E-66
ENSG00000169429	CXCL8	870	-6,82	0,30	2,2E-116	1,6E-113	886	-5,57	0,24	3,6E-121	9,2E-118
ENSG00000008853	RHOBTB2	2315	3,05	0,13	9,5E-115	6,7E-112	2081	2,84	0,14	1,3E-91	1,2E-88
ENSG00000124429	POF1B	1230	-7,13	0,33	5,1E-102	3,1E-99	1271	-3,32	0,24	3,2E-44	5,4E-42
ENSG00000108244	KRT23	2655	-6,09	0,29	2,7E-96	1,5E-93	2545	-5,96	0,26	1,1E-113	2,2E-110
ENSG00000159166	LAD1	3877	-2,62	0,13	5,2E-96	2,8E-93	4155	-1,86	0,13	6,2E-49	1,4E-46
ENSG00000139132	FGD4	2349	2,38	0,12	3,2E-95	1,6E-92	2523	2,56	0,14	3,0E-75	1,6E-72
ENSG00000186395	KRT10	14861	-3,18	0,15	5,3E-95	2,7E-92	14973	-2,94	0,16	3,4E-77	2,0E-74
ENSG00000267368	UPK3BL1	5572	-3,19	0,16	1,3E-94	6,5E-92	5616	-2,87	0,14	1,1E-93	1,1E-90
ENSG00000016602	CLCA4	796	-5,83	0,29	1,0E-91	4,8E-89	800	-4,41	0,24	2,6E-76	1,5E-73
ENSG00000164520	RAET1E	589	-5,25	0,26	1,0E-90	4,6E-88	549	-4,50	0,22	2,7E-96	3,5E-93
ENSG00000101187	SLCO4A1	1640	2,94	0,15	7,0E-83	3,0E-80	1216	2,44	0,17	1,0E-46	1,9E-44
ENSG00000197641	SERPINB13	3392	-4,16	0,22	1,3E-82	5,5E-80	3429	-3,36	0,22	3,8E-55	1,1E-52

ENSG00000142973	CYP4B1	477	-3,87	0,21	3,1E-79	1,2E-76	451	-5,11	0,25	2,4E-91	2,2E-88
ENSG00000204385	SLC44A4	479	4,85	0,26	4,8E-78	1,8E-75	716	5,49	0,22	4,8E-136	1,9E-132
ENSG00000138356	AOX1	2030	4,60	0,25	6,6E-76	2,5E-73	975	3,55	0,21	2,5E-68	1,1E-65
ENSG00000106278	PTPRZ1	663	-7,59	0,41	1,6E-75	5,7E-73	632	-6,60	0,34	9,7E-84	6,6E-81
ENSG00000213639	PPP1CB	9216	1,70	0,09	1,9E-75	6,8E-73	8182	1,53	0,10	7,2E-50	1,7E-47
ENSG00000172927	MYEOV	897	-3,53	0,19	4,6E-75	1,6E-72	940	-2,67	0,17	2,8E-57	8,6E-55
ENSG00000126947	ARMCX1	257	4,80	0,27	2,8E-71	9,2E-69	188	4,35	0,29	7,6E-53	1,9E-50
ENSG00000188089	PLA2G4E	628	-7,54	0,42	5,2E-71	1,6E-68	618	-7,22	0,39	3,2E-76	1,8E-73
ENSG00000178607	ERN1	1905	2,89	0,16	7,7E-71	2,4E-68	1389	2,39	0,15	9,6E-58	3,0E-55
ENSG00000129437	KLK14	400	-4,31	0,25	3,8E-68	1,1E-65	396	-4,63	0,25	1,3E-80	8,2E-78
ENSG00000004799	PDK4	452	6,39	0,37	3,2E-66	9,0E-64	261	5,61	0,39	3,4E-47	6,7E-45
ENSG00000206190	ATP10A	366	4,95	0,29	3,5E-64	9,7E-62	230	4,35	0,30	2,6E-47	5,2E-45
ENSG00000167757	KLK11	539	-5,21	0,31	4,8E-63	1,3E-60	555	-5,49	0,30	1,8E-74	8,9E-72
ENSG00000134352	IL6ST	12105	2,28	0,14	1,9E-62	4,8E-60	9794	1,94	0,11	1,2E-66	5,0E-64
ENSG00000170871	KIAA0232	2507	3,08	0,19	5,8E-61	1,4E-58	1607	2,33	0,16	8,2E-52	2,0E-49
ENSG00000168497	CAVIN2	1160	3,37	0,21	1,4E-60	3,2E-58	934	3,05	0,23	1,8E-41	2,7E-39
ENSG00000117114	ADGRL2	462	-5,39	0,33	1,9E-60	4,4E-58	454	-4,50	0,29	3,3E-55	9,4E-53
ENSG00000166828	SCNN1G	721	5,20	0,32	1,6E-59	3,5E-57	631	5,03	0,24	3,9E-95	4,6E-92
ENSG00000198691	ABCA4	432	-5,04	0,31	1,7E-59	3,8E-57	418	-6,09	0,38	8,8E-61	3,0E-58
ENSG00000081277	PKP1	21158	-1,94	0,12	2,0E-59	4,4E-57	21515	-1,71	0,12	5,3E-45	9,5E-43
ENSG00000171621	SPSB1	1635	3,00	0,19	2,9E-58	6,0E-56	1106	2,37	0,17	1,1E-45	2,1E-43
ENSG00000197632	SERPINB2	4633	-3,65	0,23	7,9E-58	1,5E-55	5121	-2,05	0,13	3,6E-54	9,3E-52
ENSG00000143556	S100A7	6078	-7,49	0,48	5,9E-57	1,1E-54	6000	-9,82	0,50	2,0E-87	1,8E-84
ENSG00000184012	TMPRSS2	697	4,40	0,28	1,9E-55	3,4E-53	496	3,94	0,20	1,0E-85	7,8E-83
ENSG00000087495	PHACTR3	621	4,46	0,31	1,0E-49	1,6E-47	441	3,98	0,28	5,0E-47	9,6E-45
