# Supplementary material for: Nonmalignant AR-positive prostate epithelial cells and cancer cells respond differently to androgen
Source: Endocr Relat Cancer. 2022 Oct 10;29(12):717–33. doi: 10.1530/ERC-22-0108 (PMC9644224; doi:10.1530/ERC-22-0108)
Supplement: Supplementary table 4. List of mutually upregulated genes in RWPE-1-AR clones and LNCaP-pcDNA3.1 in 100 vs 0 nM DHT. [file supplementary_table_4.pdf]

Supplementary table 4. List of mutually upregulated genes in RWPE-1-AR clones and LNCaP-pcDNA3.1 in 100 vs 0 nM DHT.

		RWPE-1-AR clones 100 vs 0 nM DHT					LNCaP-pcDNA3.1 100 vs 0 nM DHT				
Ensemble gene id	Hgnc symbol	baseMean	log_2FC	lfcSE	P	P_{adj}	baseMean	log_2FC	lfcSE	P	P_{adj}
ENSG00000096060	FKBP5	6761	3,02	0,20	2,12E-53	2,54E-50	2473	1,14	0,32	1,68E-05	0,00210
ENSG00000109906	ZBTB16	335	5,35	0,37	1,23E-49	1,12E-46	566	1,93	0,18	5,17E-27	1,59E-23
ENSG00000116574	RHOU	419	2,38	0,33	3,39E-14	2,32E-12	2338	1,86	0,20	2,79E-22	4,76E-19
ENSG00000178573	MAF	229	1,54	0,21	4,16E-15	3,22E-13	748	2,29	0,77	8,84E-05	0,00900
ENSG00000184012	TMPRSS2	746	1,39	0,24	6,18E-10	2,41E-08	2355	2,32	0,21	3,34E-30	1,28E-26
ENSG00000196208	GREB1	42	1,73	0,42	2,00E-06	3,82E-05	675	1,72	0,23	1,34E-15	1,29E-12
ENSG00000221869	CEBPD	1263	1,36	0,17	1,38E-17	1,39E-15	460	1,25	0,21	5,72E-11	2,51E-08
