# Supplementary material for: Nonmalignant AR-positive prostate epithelial cells and cancer cells respond differently to androgen
Source: Endocr Relat Cancer. 2022 Oct 10;29(12):717–33. doi: 10.1530/ERC-22-0108 (PMC9644224; doi:10.1530/ERC-22-0108)
Supplement: Supplementary table 5. List of mutually upregulated genes in RWPE-1-AR clones and LNCaP-ARhi in 100 vs 0 nM DHT. [file supplementary_table_5.pdf]

Supplementary table 5. List of mutually upregulated genes in RWPE-1-AR clones and LNCaP-ARhi in 100 vs 0 nM DHT.

		RWPE-1-AR clones 100 vs 0 nM DHT					LNCaP-ARhi 100 vs 0 nM DHT				
Ensemble gene id	Hgnc symbol	baseMean	log_2FC	lfcSE	P	P_{adj}	baseMean	log_2FC	lfcSE	P	P_{adj}
ENSG00000118515	SGK1	1791	2,77	0,08	1,06E-234	1,64E-230	885	4,92	0,20	6,42E-138	2,41E-134
ENSG00000163659	TIPARP	3836	1,59	0,16	5,95E-24	1,22E-21	1420	3,78	0,16	2,17E-132	6,52E-129
ENSG00000116574	RHOU	419	2,38	0,33	3,39E-14	2,32E-12	3077	3,71	0,16	3,41E-127	8,53E-124
ENSG00000178573	MAF	229	1,54	0,21	4,16E-15	3,22E-13	1403	3,57	0,19	2,40E-80	3,00E-77
ENSG00000184012	TMPRSS2	746	1,39	0,24	6,18E-10	2,41E-08	5332	3,54	0,23	3,55E-53	2,53E-50
ENSG00000116285	ERRFI1	1944	1,31	0,22	1,03E-10	4,55E-09	3095	3,34	0,13	4,56E-151	2,28E-147
ENSG00000096060	FKBP5	6761	3,02	0,20	2,12E-53	2,54E-50	6181	3,29	0,15	9,43E-108	2,02E-104
ENSG00000156284	CLDN8	387	2,31	0,22	4,96E-28	1,40E-25	192	3,22	0,23	2,21E-45	1,10E-42
ENSG00000204682	MIR1915HG	158	1,92	0,26	1,91E-14	1,36E-12	75	3,12	0,33	4,84E-22	7,98E-20
ENSG00000109906	ZBTB16	335	5,35	0,37	1,23E-49	1,12E-46	855	3,08	0,20	1,02E-52	6,96E-50
ENSG00000152086	TUBA3E	3	2,80	1,31	0,00139	0,0119	14	2,81	0,97	0,000161	0,00407
ENSG00000104419	NDRG1	11850	1,61	0,26	6,75E-11	3,09E-09	6127	2,71	0,24	1,27E-31	3,54E-29
ENSG00000175040	CHST2	2108	3,66	0,41	1,33E-20	1,97E-18	29	2,61	0,60	5,64E-07	2,53E-05
ENSG00000144749	LRIG1	219	1,03	0,20	2,15E-08	6,24E-07	3172	2,38	0,12	8,10E-85	1,10E-81
ENSG00000178607	ERN1	1630	2,42	0,19	9,56E-38	4,96E-35	1525	1,97	0,14	8,80E-44	4,26E-41
ENSG00000221869	CEBPD	1263	1,36	0,17	1,38E-17	1,39E-15	430	1,94	0,15	3,10E-41	1,33E-38
ENSG00000171621	SPSB1	1489	1,74	0,18	1,97E-22	3,67E-20	191	1,86	0,29	4,27E-12	3,50E-10
ENSG00000196208	GREB1	42	1,73	0,42	2,00E-06	3,82E-05	908	1,73	0,13	1,70E-41	7,49E-39
ENSG00000139132	FGD4	2643	1,63	0,10	9,78E-66	1,52E-62	559	1,51	0,26	2,87E-10	2,00E-08
ENSG00000095397	WHRN	221	1,11	0,27	2,61E-06	4,75E-05	541	1,37	0,22	3,35E-11	2,50E-09
ENSG00000113083	LOX	254	1,33	0,26	1,80E-08	5,30E-07	693	1,29	0,24	4,57E-09	2,79E-07
ENSG00000123700	KCNJ2	23	1,31	0,30	6,37E-07	1,36E-05	35	1,27	0,53	0,000621	0,0135
ENSG00000163884	KLF15	387	2,55	0,31	3,57E-18	3,81E-16	696	1,20	0,13	3,81E-22	6,35E-20
ENSG00000204103	MAFB	597	2,47	0,21	7,22E-33	2,81E-30	1928	1,20	0,14	3,06E-19	4,09E-17
ENSG00000135686	KLHL36	2043	1,07	0,18	1,12E-10	4,88E-09	1605	1,17	0,21	1,05E-09	6,99E-08
ENSG00000117586	TNFSF4	16	1,28	0,37	2,47E-05	0,000360	109	1,14	0,26	9,20E-07	3,94E-05
ENSG00000067113	PLPP1	998	1,73	0,14	1,68E-38	9,32E-36	5299	1,14	0,11	6,03E-25	1,16E-22
ENSG00000136870	ZNF189	638	1,25	0,17	6,68E-15	5,09E-13	1214	1,12	0,26	1,22E-06	5,01E-05
ENSG00000026559	KCNG1	204	1,09	0,21	1,46E-08	4,36E-07	61	1,03	0,43	0,000729	0,0155
