# Supplementary material for: Nonmalignant AR-positive prostate epithelial cells and cancer cells respond differently to androgen
Source: Endocr Relat Cancer. 2022 Oct 10;29(12):717–33. doi: 10.1530/ERC-22-0108 (PMC9644224; doi:10.1530/ERC-22-0108)
Supplement: Supplementary table 6. List of mutually downregulated genes in RWPE-1-AR clones and LNCaP-ARhi in 100 vs 0 nM DHT. [file supplementary_table_6.pdf]

Supplementary table 6. List of mutually downregulated genes in RWPE-1-AR clones and LNCaP-ARhi in 100 vs 0 nM DHT.

		RWPE-1-AR clones 100 vs 0 nM DHT					LNCaP-ARhi 100 vs 0 nM DHT				
Ensemble gene id	Hgnc symbol	baseMean	log_2FC	lfcSE	P	P_{adj}	baseMean	log_2FC	lfcSE	P	P_{adj}
ENSG00000171617	ENC1	3357	-1,45	0,26	1,36E-09	5,01E-08	1111	-1,06	0,25	1,46E-06	5,93E-05
ENSG00000162009	SSTR5	25	-1,09	0,43	0,000504	0,00500	266	-1,25	0,20	1,10E-11	8,72E-10
ENSG00000181634	TNFSF15	1562	-1,62	0,20	9,77E-17	8,94E-15	260	-1,80	0,22	1,76E-17	2,11E-15
ENSG00000095739	BAMBI	301	-1,29	0,21	2,46E-11	1,20E-09	553	-1,97	0,18	1,68E-30	4,49E-28
ENSG00000139211	AMIGO2	662	-2,12	0,23	1,30E-21	2,20E-19	81	-2,74	0,31	1,33E-19	1,81E-17
