# Supplementary material for: Nonmalignant AR-positive prostate epithelial cells and cancer cells respond differently to androgen
Source: Endocr Relat Cancer. 2022 Oct 10;29(12):717–33. doi: 10.1530/ERC-22-0108 (PMC9644224; doi:10.1530/ERC-22-0108)
Supplement: Supplementary table 7. Significantly enriched gene sets in 1 nM DHT vs 0 nM DHT in RWPE-1-ARc5. [file supplementary_table_7.pdf]

Supplementary table 7. Significantly enriched gene sets in 1 nM DHT vs 0 nM DHT in RWPE-1-ARc5.

pathway	P	P_{adj}	ES	NES	nMoreExtreme	size
HALLMARK_KRAS_SIGNALING_DN	0,00147	0,0256	0,653	1,87	0	139
HALLMARK_ANDROGEN_RESPONSE	0,00154	0,0256	0,641	1,76	0	95
HALLMARK_HYPOXIA	0,00138	0,0256	0,571	1,69	0	185
HALLMARK_INTERFERON_ALPHA_RESPONSE	0,00279	0,0348	-0,580	-1,76	0	93
HALLMARK_ESTROGEN_RESPONSE_LATE	0,00421	0,0421	0,518	1,52	2	178
HALLMARK_INTERFERON_GAMMA_RESPONSE	0,0105	0,0874	-0,449	-1,48	2	179
