# Supplementary material for: Nonmalignant AR-positive prostate epithelial cells and cancer cells respond differently to androgen
Source: Endocr Relat Cancer. 2022 Oct 10;29(12):717–33. doi: 10.1530/ERC-22-0108 (PMC9644224; doi:10.1530/ERC-22-0108)
Supplement: Supplementary table 8. Significantly enriched gene sets in 100 nM DHT vs 0 nM DHT in RWPE-1-ARc5. [file supplementary_table_8.pdf]

Supplementary table 8. Significantly enriched gene sets in 100 nM DHT vs 0 nM DHT in RWPE-1-ARc5.

pathway	P	P_{adj}	ES	NES	nMoreExtreme	size
HALLMARK_INTERFERON_ALPHA_RESPONSE	0,00248	0,0267	-0,618	-1,91	0	94
HALLMARK_INTERFERON_GAMMA_RESPONSE	0,00274	0,0267	-0,512	-1,74	0	180
HALLMARK_KRAS_SIGNALING_DN	0,00161	0,0267	0,570	1,72	0	139
HALLMARK_KRAS_SIGNALING_UP	0,00270	0,0267	-0,465	-1,54	0	158
HALLMARK_TNFA_SIGNALING_VIA_NFKB	0,00265	0,0267	-0,440	-1,51	0	184
HALLMARK_HYPOXIA	0,00320	0,0267	0,485	1,50	1	184
HALLMARK_INFLAMMATORY_RESPONSE	0,00525	0,0375	-0,453	-1,51	1	155
HALLMARK_ALLOGRAFT_REJECTION	0,00792	0,0495	-0,454	-1,49	2	140
HALLMARK_ANDROGEN_RESPONSE	0,0100	0,0539	0,542	1,57	5	95
