# Supplementary material for: Nonmalignant AR-positive prostate epithelial cells and cancer cells respond differently to androgen
Source: Endocr Relat Cancer. 2022 Oct 10;29(12):717–33. doi: 10.1530/ERC-22-0108 (PMC9644224; doi:10.1530/ERC-22-0108)
Supplement: Supplementary table 9. Significantly enriched genesets in 1 nM vs 0 nm DHT in RWPE-1-ARc15. [file supplementary_table_9.pdf]

Supplementary table 9. Significantly enriched genesets in 1 nM vs 0 nm DHT in RWPE-1-ARc15.

pathway	P	P_{adj}	ES	NES	nMoreExtreme	size
HALLMARK_KRAS_SIGNALING_DN	0,00134	0,0234	0,705	1,99	0	137
HALLMARK_ANDROGEN_RESPONSE	0,00140	0,0234	0,685	1,86	0	95
HALLMARK_ESTROGEN_RESPONSE_LATE	0,00128	0,0234	0,598	1,74	0	178
HALLMARK_HYPOXIA	0,00253	0,0316	0,557	1,63	1	186
HALLMARK_INTERFERON_ALPHA_RESPONSE	0,0201	0,168	-0,508	-1,59	5	93
