# Supplementary material for: Nonmalignant AR-positive prostate epithelial cells and cancer cells respond differently to androgen
Source: Endocr Relat Cancer. 2022 Oct 10;29(12):717–33. doi: 10.1530/ERC-22-0108 (PMC9644224; doi:10.1530/ERC-22-0108)
Supplement: Supplementary table 10. Significantly enriched gene sets in 100 nM vs 0 nm DHT in RWPE-1-ARc15. [file supplementary_table_10.pdf]

Supplementary table 10. Significantly enriched gene sets in 100 nM vs 0 nm DHT in RWPE-1-ARc15.

pathway	P	P_{adj}	ES	NES	nMoreExtreme	size
HALLMARK_KRAS_SIGNALING_DN	0,00140	0,0248	0,66	1,96	0	136
HALLMARK_INTERFERON_ALPHA_RESPONSE	0,00298	0,0248	-0,575	-1,84	0	93
HALLMARK_ANDROGEN_RESPONSE	0,00149	0,0248	0,636	1,81	0	94
HALLMARK_ESTROGEN_RESPONSE_LATE	0,00133	0,0248	0,534	1,65	0	178
HALLMARK_HYPOXIA	0,00269	0,0248	0,529	1,63	1	185
HALLMARK_INTERFERON_GAMMA_RESPONSE	0,00397	0,0248	-0,456	-1,58	0	180
HALLMARK_KRAS_SIGNALING_UP	0,00364	0,0248	-0,447	-1,54	0	160
HALLMARK_TNFA_SIGNALING_VIA_NFKB	0,00383	0,0248	-0,431	-1,51	0	183
HALLMARK_ALLOGRAFT_REJECTION	0,00694	0,0358	-0,436	-1,47	1	139
HALLMARK_INFLAMMATORY_RESPONSE	0,00717	0,0358	-0,408	-1,40	1	154
