# Supplementary material for: Nonmalignant AR-positive prostate epithelial cells and cancer cells respond differently to androgen
Source: Endocr Relat Cancer. 2022 Oct 10;29(12):717–33. doi: 10.1530/ERC-22-0108 (PMC9644224; doi:10.1530/ERC-22-0108)
Supplement: Supplementary table 11. Comparison of RWPE-1-ARc5 and ARc15 cells against RWPE-1-Ctrlc1 in 0 nM DHT. [file supplementary_table_11.pdf]

Supplementary table 11. Comparison of RWPE-1-ARc5 and ARc15 cells against RWPE-1-Ctrlc1 in 0 nM DHT.

pathway	P	P_{adj}	ES	NES	nMoreExtreme	size
HALLMARK_E2F_TARGETS	0,00129	0,0156	-0,666	-2,24	0	198
HALLMARK_MYC_TARGETS_V1	0,00129	0,0156	-0,653	-2,19	0	195
HALLMARK_G2M_CHECKPOINT	0,00129	0,0156	-0,587	-1,96	0	193
HALLMARK_MYC_TARGETS_V2	0,00156	0,0156	-0,664	-1,88	0	58
HALLMARK_KRAS_SIGNALING_DN	0,00135	0,0156	-0,528	-1,71	0	149
HALLMARK_INTERFERON_GAMMA_RESPONSE	0,00418	0,0299	0,413	1,61	0	185
HALLMARK_ESTROGEN_RESPONSE_LATE	0,00393	0,0299	-0,470	-1,56	2	182
HALLMARK_IL6_JAK_STAT3_SIGNALING	0,00898	0,0449	0,471	1,63	2	74
HALLMARK_INFLAMMATORY_RESPONSE	0,00760	0,0449	0,383	1,47	1	165
HALLMARK_HYPOXIA	0,00881	0,0449	0,355	1,38	1	188
HALLMARK_ALLOGRAFT_REJECTION	0,0112	0,0509	0,391	1,49	2	153
