# Supplementary material for: Nonmalignant AR-positive prostate epithelial cells and cancer cells respond differently to androgen
Source: Endocr Relat Cancer. 2022 Oct 10;29(12):717–33. doi: 10.1530/ERC-22-0108 (PMC9644224; doi:10.1530/ERC-22-0108)
Supplement: Supplementary table 12. Significantly enriched gene sets in 100 nM vs 0 nM DHT in LNCaP-pcDNA3.1 [file supplementary_table_12.pdf]

Supplementary table 12. Significantly enriched gene sets in 100 nM vs 0 nM DHT in LNCaP-pcDNA3.1

pathway	P	P_{adj}	ES	NES	nMoreExtreme	size
HALLMARK_ANDROGEN_RESPONSE	0,00124	0,062	0,939	1,71	0	98
HALLMARK_EPITHELIAL_MESENCHYMAL_TRANSITION	0,00936	0,234	0,818	1,52	7	140
