# Supplementary material for: Nonmalignant AR-positive prostate epithelial cells and cancer cells respond differently to androgen
Source: Endocr Relat Cancer. 2022 Oct 10;29(12):717–33. doi: 10.1530/ERC-22-0108 (PMC9644224; doi:10.1530/ERC-22-0108)
Supplement: Supplementary table 13. Significantly enriched genes sets in 100 vs 0 nM DHT in LNCaP-ARhi. [file supplementary_table_13.pdf]

Supplementary table 13. Significantly enriched genes sets in 100 vs 0 nM DHT in LNCaP-ARhi.

pathway	P	P_{adj}	ES	NES	nMoreExtreme	size
HALLMARK_ANDROGEN_RESPONSE	0,00122	0,0122	0,910	2,20	0	98
HALLMARK_EPITHELIAL_MESENCHYMAL_TRANSITION	0,00113	0,0122	0,725	1,81	0	141
HALLMARK_HYPOXIA	0,00109	0,0122	0,708	1,78	0	184
HALLMARK_TNFA_SIGNALING_VIA_NFKB	0,00111	0,0122	0,708	1,78	0	168
HALLMARK_KRAS_SIGNALING_UP	0,00114	0,0122	0,710	1,77	0	135
HALLMARK_MYC_TARGETS_V2	0,00380	0,0317	-0,625	-1,82	0	58
HALLMARK_KRAS_SIGNALING_DN	0,00680	0,0485	0,628	1,57	5	141
