# Supplementary material for: Nonmalignant AR-positive prostate epithelial cells and cancer cells respond differently to androgen
Source: Endocr Relat Cancer. 2022 Oct 10;29(12):717–33. doi: 10.1530/ERC-22-0108 (PMC9644224; doi:10.1530/ERC-22-0108)
Supplement: Supplementary table 14. Significantly enriched gene sets in 1 nM vs 0 nM DHT in LNCaP-ARhi. [file supplementary_table_14.pdf]

Supplementary table 14. Significantly enriched gene sets in 1 nM vs 0 nM DHT in LNCaP-ARhi.

| pathway | P | $P_{adj}$ | ES | NES | nMoreExtreme | size |
|---|---|---|---|---|---|---|
| HALLMARK_ANDROGEN_RESPONSE | 0,00129 | 0,0322 | 0,946 | 1,76 | 0 | 98 |
| HALLMARK_EPITHELIAL_MESENCHYMAL_TRANSITION | 0,00122 | 0,0322 | 0,900 | 1,69 | 0 | 138 |
| HALLMARK_MTORC1_SIGNALING | 0,00862 | 0,0870 | -0,644 | -1,96 | 0 | 194 |
