# Supplementary material for: Nonmalignant AR-positive prostate epithelial cells and cancer cells respond differently to androgen
Source: Endocr Relat Cancer. 2022 Oct 10;29(12):717–33. doi: 10.1530/ERC-22-0108 (PMC9644224; doi:10.1530/ERC-22-0108)
Supplement: Supplementary table 15. List of primary antibodies. [file supplementary_table_15.pdf]

Supplementary table 15. List of primary antibodies.

antibody name / target	manufacturer	dilution for WB	dilution for fluorescence microscopy
mouse α-AR 441	Neomarkers/Thermo Fisher Scientific	1:1000	1:100
rabbit α-ERK1/2 (EPR17526)	Abcam, Cambridge, UK	1:10000	-
rabbit α-ERK1 (phospho T202) + ERK2 (phospho T185) (EPR19401)	Abcam, Cambridge, UK	1:500	-
mouse α-CDK4 (DCS156)	Cell Signaling Technology, Danvers, MA, USA	1:2000	-
mouse α-lamin B	Invitrogen/Thermo Fisher Scientific	1:1000	-
rabbit α-lamin B	Invitrogen/Thermo Fisher Scientific	1:1000	
mouse α-β-tubulin	Sigma-Aldrich / Merck Millipore	1:4000	
mouse α-Ki-67	Novocastra/Leica Biosystems, Wetzlar, Germany	-	1:200
