# Supplementary material for: Nonmalignant AR-positive prostate epithelial cells and cancer cells respond differently to androgen
Source: Endocr Relat Cancer. 2022 Oct 10;29(12):717–33. doi: 10.1530/ERC-22-0108 (PMC9644224; doi:10.1530/ERC-22-0108)
Supplement: Supplementary table 16. List of primers. [file supplementary_table_16.pdf]

Supplementary table 16. List of primers.

primer	sequence	PrimerBank ID
TMPRSS2 cDNA for	GCAGTGGTTTCTTTACGCTGT	227499989c3
TMPRSS2 cDNA rev	CCGCAAATGCCGTCCAATG	227499989c3
FKBP5 cDNA for	CGGCGACAGGTTCTCTACTT	
FKBP5 cDNA rev	CATGGTAGCCACCCCAATGT	
AR cDNA for	CTTTGCAGCCTTGCTCTCTA	
AR cDNA rev	TCTGGTCGTCCACGTGTAA	
NKX3-1 cDNA for	GAGACGCTGGCAGAGACC	
NKX3-1 cDNA rev	CACCTGAGTGTGGGAGAAGG	
KLK3_cDNA for	CCAAGTTCATGCTGTGTGCT	
KLK3_cDNA rev	CCCATGACGTGATACCTTGA	
KRT8 cDNA for	CAGAAGTCCTACAAGGTGTCCA	372466576c1
KRT8 cDNA rev	CTCTGGTTGACCGTAACTGCG	372466576c1
KRT5 cDNA for	TTGGACCAGTCAACATCTCTGT	
KRT5 cDNA rev	ACTGCTACCTCCGGCAAGAC	
TBP_for	GAATATAATCCCAAGCGGTTTG	
TBP_rev	ACTTCACATCACAGCTCCCC	
